# An Expanded Toolbox for Versatile Chemical Editing of Adeno‐Associated Virus

**DOI:** 10.1002/anie.202516157

**Published:** 2026-01-01

**Authors:** Quan Pham, Jake Glicksman, Boyang Han, David Koo, Conor Loynd, Soumya Jyoti Singha Roy, Abhishek Chatterjee

**Affiliations:** ^1^ Department of Chemistry Boston College 2609 Beacon Street Chestnut Hill Massachusetts 02467 USA

**Keywords:** AAV engineering, Bioorthogonal chemistry, Gene therapy, Genetic code expansion, Noncanonical amino acids

## Abstract

Site‐specific incorporation of noncanonical amino acids (ncAAs) into the adeno‐associated virus (AAV) capsid offers powerful opportunities to probe and engineer the properties of this leading vector for human gene therapy. However, this approach currently relies almost exclusively on a single azide‐containing ncAA, incorporated using the pyrrolysyl‐tRNA synthetase/tRNA pair. Here, we substantially broaden the scope of this technology by demonstrating successful incorporation of numerous ncAAs into AAV capsid using four different platforms, and by uncovering design principles that facilitate capsid tolerance to structurally diverse side chains. Using this expanded toolbox, we incorporate several different bioorthogonal conjugation handles into AAV for precise capsid modification. In particular, a tetrazine‐containing ncAA facilitated ultrafast conjugation of an anti‐HER2 nanobody to the capsid, creating conjugates that efficiently and selectively infect HER2+ cells. We further used this platform for optimized capsid PEGylation, which reduced its immunogenicity without compromising infectivity. Finally, we efficiently incorporated two distinct ncAAs into the AAV capsid, and subsequently labeled them orthogonally to attach two different entities. Together, these advances dramatically expand the chemistries that can be introduced into the AAV capsid, offering powerful new tools to both probe and engineer the properties of this promising gene therapy vector.

## Graphical abstract

We describe technology to introduce diverse non‐natural chemical functionalities site‐specifically into the capsid of adeno‐associated virus through genetic code expansion, and using them to engineer this leading vector for human gene therapy for enhanced tissue specificity and reduced immunogenicity

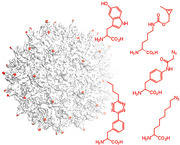

## Introduction

AAV is a non‐enveloped parvovirus, harboring a ∼4.7 kb single‐stranded DNA genome, which encodes three capsid proteins VP1, VP2, and VP3, that are incorporated into its icosahedral capsid in approximately 1:1:10 ratio. Over the last decade, it has emerged as the leading candidate for in vivo human gene therapy, empowering the development of several FDA‐approved therapeutics, including Luxturna, Zolgensma, and Hemgenix, etc.^[^ ^1^, ^2^, ^3^, ^4^, ^5^ ^]^ Despite its remarkable clinical success, wild‐type AAV capsids, which nearly all approved gene therapies and ongoing clinical trials rely on, suffer from critical limitations such as suboptimal tissue tropism, and adaptive immune response that prevents repeat dosing.^[^ ^1^, ^2^, ^3^, ^4^, ^5^, ^6^ ^]^

Considerable effort has been devoted to overcoming these limitations through AAV capsid engineering. For example, directed evolution — using approaches such as error‐prone PCR, DNA shuffling, or randomized peptide insertion — has yielded novel variants with altered tissue tropism.^[^ ^7^, ^8^, ^9^, ^10^, ^11^, ^12^, ^13^, ^14^, ^15^, ^16^, ^17^, ^18^, ^19^, ^20^, ^21^ ^]^ However, this strategy is time‐consuming and labor‐intensive, and the enhanced properties of the evolved capsids identified in vitro, or in animal models, often fail to translate across preclinical systems. Rational engineering of the capsid by fusing retargeting peptides or proteins onto specific capsid proteins has also been used.^[^ ^22^, ^23^, ^24^, ^25^, ^26^, ^27^, ^28^, ^29^ ^]^ However, this method is constrained to small and well‐folded proteins due to the fusion‐associated perturbation of the capsid. Fusion onto the capsid proteins also runs the risk of disrupting the packaging efficiency and structural integrity of the capsid, which may result in diminished titer and suboptimal infectivity.

Chemical modification of the capsid represents an alternative approach to AAV engineering. Modification of surface‐exposed canonical amino acid residues such lysine, arginine, and tyrosine has been used to both investigate and engineer the properties of AAV.^[^ ^4^, ^30^, ^31^, ^32^, ^33^, ^34^, ^35^ ^]^ However, relying on canonical amino acid residues is intrinsically associated with limited control over the site and stoichiometry of capsid modification. The use of genetic code expansion (GCE) technology,^[^ ^36^, ^37^, ^38^, ^39^ ^]^ which enables site‐specific incorporation of bioorthogonal noncanonical amino acids (ncAA) into proteins using engineered aminoacyl‐tRNA synthetase (aaRS)/tRNA pairs, offers a more precise alternative for chemical capsid modification.^[^ ^4^, ^40^, ^41^, ^42^, ^43^, ^44^, ^45^, ^46^, ^47^, ^48^, ^49^, ^50^ ^]^

**Figure 1:**
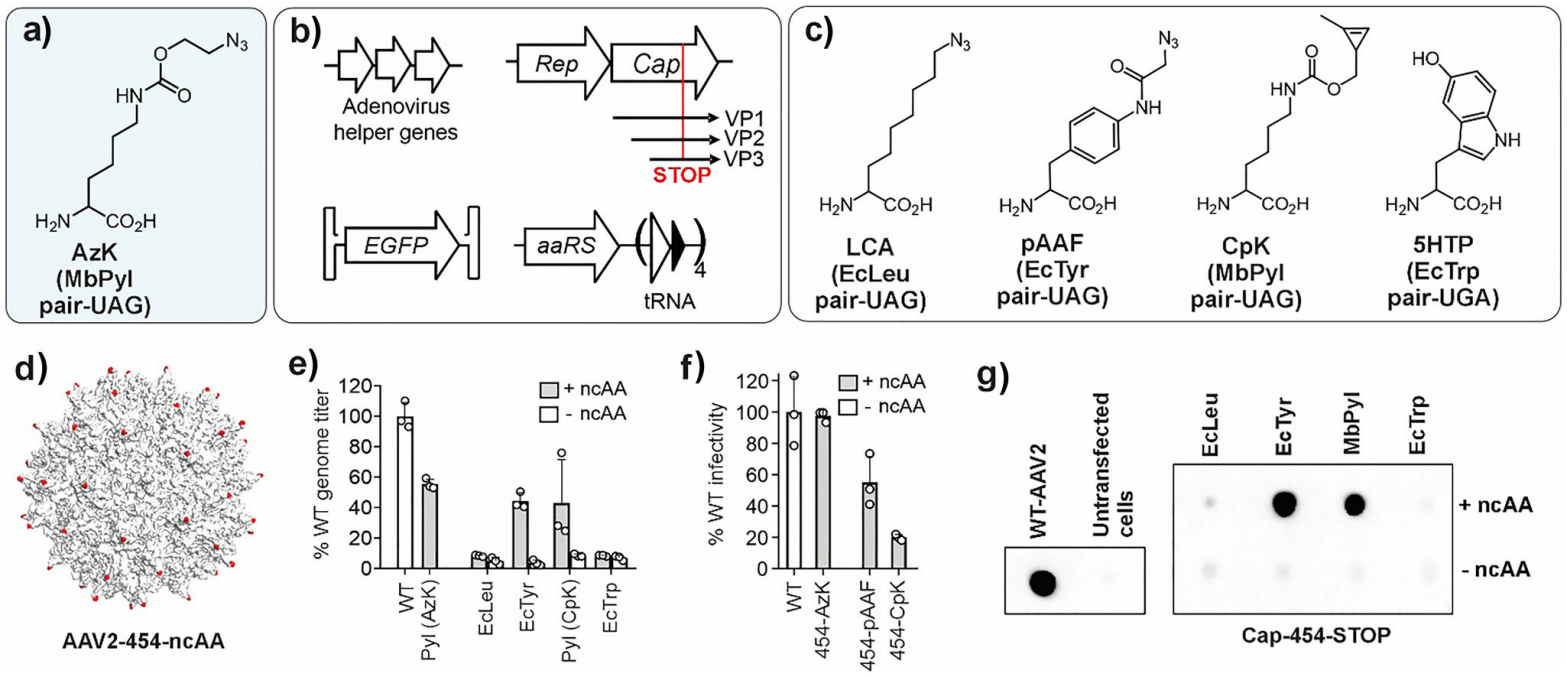
Incorporation of different ncAAs in all capsid proteins of AAV. a) Structure of AzK. b) Elements for packaging ncAA‐containing AAV2. c) Structure of ncAAs tested here, the corresponding aaRS/tRNA pairs, and the stop codon these suppress. d) Structure of the AAV2 capsid showing the distribution of the surface‐exposed T454 residue (PDB ID: 6IH9). e) Production of various ncAA‐mutants of AAV2 (packaged genome copies measured by qPCR) in the presence or absence of ncAA in the media, normalized to the % of WT AAV2 titer. f) Infectivity of the AzK‐, pAAF‐, and CpK‐mutants of AAV2, normalized to the % infectivity of WT AAV2, measured by the expression of an encoded EGFP reporter, upon infecting HEK293T cells at a constant MOI 50 (see Figure S1). Data shown as the mean ± s.d. of *n* =  3 replicates. g) Dot‐blot analysis for the production of packaged capsids of WT AAV2 and various ncAA‐mutants in cell‐free extract, using anti‐AAV antibody (clone A20).

Hundreds of different ncAAs can now be incorporated into proteins expressed in mammalian cells, using multiple aaRS/tRNA pairs, which offer powerful new ways to probe and manipulate protein function.^[^ ^36^, ^37^, ^38^, ^39^ ^]^ However, the application of GCE for AAV has relied exclusively on the pyrrolysyl‐tRNA synthetase (PylRS)/tRNA pair^[^ ^51^ ^]^ to incorporate the bioorthogonal ncAA AzK (Figure 1a).^[^ ^4^, ^40^, ^41^, ^42^, ^43^, ^44^, ^45^, ^46^, ^47^, ^48^, ^49^, ^50^ ^]^ Here, we demonstrate that this limitation stems from the sensitivity of the AAV capsid to the incorporation of many ncAAs into all 60 capsid proteins (VP1, VP2, and VP3). However, when ncAA incorporation is targeted selectively to the minor capsid proteins, a much broader spectrum of ncAAs can be successfully incorporated without compromising virus titer or infectivity. This finding is particularly significant in light of our recent demonstration that optimal AAV engineering is achieved through controlled, low‐stoichiometry modifications (5–10 per capsid) rather than more extensive capsid labeling, which disrupts capsid function.^[^ ^41^, ^45^ ^]^ Thus, the ability to incorporate diverse ncAAs into minor capsid proteins not only circumvents the inherent sensitivity of the capsid but also aligns with the optimal modification parameters for functional AAV conjugates.

Using this strategy, we successfully incorporated numerous ncAAs into the AAV capsid with diverse sidechain chemistries and architectures, employing four different aaRS/tRNA pairs. We incorporated multiple bioconjugation handles, including azides, alkynes, strained alkenes, ketones, 5‐hydroxytryptophan, tetrazines, and demonstrated their use for site‐specific capsid modification. Incorporation of the tetrazine functionality enabled the use of ultrafast inverse‐electron demand Diels‐Alder reaction (IEDDA)^[^ ^52^, ^53^, ^54^ ^]^ to directly conjugate a HER2‐targeting nanobody, allowing efficient retargeting to HER2+ cancer cells. We also optimized the site and stoichiometry of PEG polymer labeling to preserve infectivity while significantly reducing antibody responses in immune‐competent mice. Finally, we demonstrate for the first time the simultaneous incorporation of two different ncAAs into one AAV capsid using orthogonal aaRS/tRNA pairs that read distinct nonsense codons. This technology enabled labeling of different capsid proteins with mutually orthogonal bioconjugation chemistries for distinct payload attachment. Taken together, our work remarkably expands the scope of GCE technology for AAV engineering by broadening both the types of ncAAs that can be incorporated, and the versatility of capsid modification, while providing proof‐of‐concept demonstrations for engineering AAV properties, including tissue targeting and reduced immunogenicity.

## Results and Discussion

### ncAA Mutagenesis of All Capsid Proteins is Poorly Tolerated

To expand the GCE toolbox for AAV engineering beyond AzK, we sought to incorporate ncAAs with diverse sidechains and chemical handles that had been successfully incorporated into proteins in mammalian cells. To this end, we used our previously established 3‐plasmid transfection system,^[^ ^40^, ^41^, ^43^, ^44^, ^45^ ^]^ comprising A) adenovirus helper genes, B) AAV‐genes Rep and nonsense mutant of Cap, C) AAV genome encoding an GFP reporter flanked by inverted terminal repeats (ITRs), and D) an ncAA‐selective orthogonal aaRS/tRNA pair (Figure 1b). An amber or opal stop codon was introduced at the T454 site of the Cap gene, which would result in ncAA incorporation across all 60 capsid proteins (VP1, VP2, and VP3) (Figure 1d). This surface‐exposed position was chosen based on prior studies demonstrating its tolerance for ncAA incorporation.^[^ ^41^, ^45^ ^]^ Specifically, a TAG codon was used for incorporating (Figure 1c): 1) LCA using an engineered *E. coli* leucyl‐tRNA synthetase (EcLeuRS)/tRNA_CUA_ pair;^[^ ^55^, ^56^, ^57^ ^]^ 2) CpK using *M. barkeri* PylRS/tRNA_CUA_ pair;^[^ ^58^, ^59^, ^60^ ^]^ 3) pAAF employing an engineered *E. coli* tyrosyl‐tRNA synthetase (EcTyrRS)/tRNA_CUA_ pair.^[^ ^61^ ^]^ For incorporating 5‐hydroxytryptophan (5HTP), T454 was mutated to a TGA codon, and suppressed by an engineered *E. coli* tryptophanyl‐tRNA synthetase (EcTrpRS)/tRNA_UCA_ pair.^[^ ^62^, ^63^ ^]^ For this particular application, TGA stop codons in native AAV genes were mutated to TAA to ensure proper termination of these proteins.

Each AAV packaging experiment was performed in the presence or absence of the ncAA substrate. Only in the presence of ncAA, the capsid proteins should be expressed through nonsense suppression and successful AAV production should be observed. Analysis of packaged genomes by qPCR showed successful virus production with pAAF and CpK, each yielding approximately 40% of wild‐type levels, but not with LCA and 5HTP (Figure 1e). These observations were corroborated by dot‐blot analysis using an anti‐AAV2 antibody (A20 clone) that selectively binds intact capsids (Figure 1g). Although both T454‐pAAF and T454‐CpK mutants were successfully packaged, each showed significantly attenuated infectivity relative to wild‐type AAV2 when tested on HEK293T cells at a constant multiplicity of infection (MOI; genome copies/cell) of 50 (Figures 1f, S1). These observations suggest that the tolerance of AAV capsid for AzK incorporation is somewhat unique. It exhibits substantial sensitivity to the incorporation of other ncAAs across all 60 capsid proteins, which restricts the scope of GCE technology for AAV engineering.

### Successful Incorporation of Diverse ncAAs Into Minor Capsid Proteins

**Figure 2:**
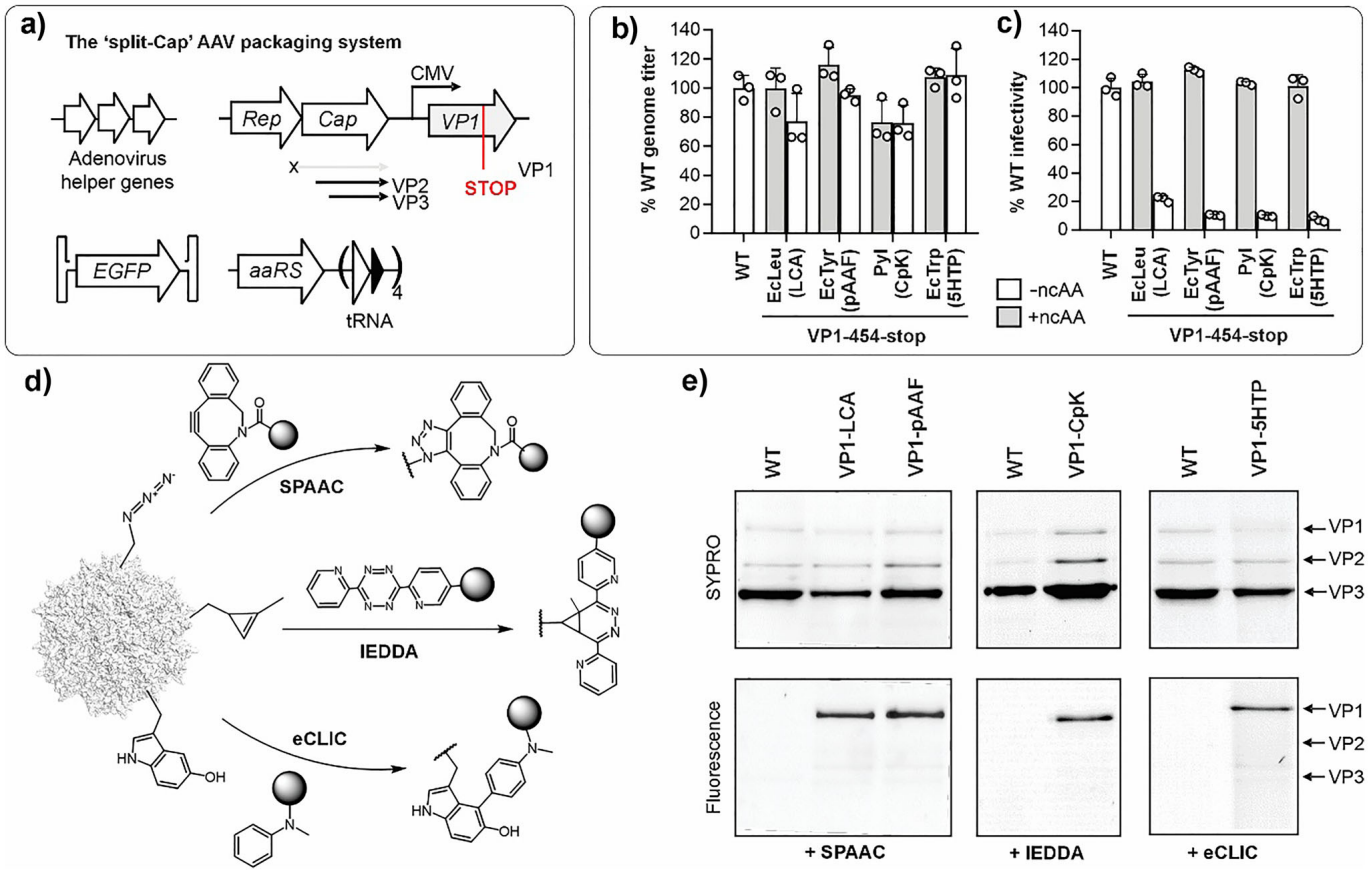
Incorporation of different ncAAs into VP1 alone. a) The “split‐Cap” plasmid system for producing AAV2 selectively incorporating into VP1. b) Production of various VP1‐454‐ncAA mutants of AAV2 (packaged genome copies measured by qPCR) in the presence or absence of ncAA, normalized to the % of WT AAV2 titer. c) Infectivity of various VP1‐454‐ncAA mutants of AAV2, normalized to the % infectivity of WT AAV2, measured by the expression of an encoded EGFP reporter, upon infecting HEK293T cells at a constant MOI 50 (see Figure S2). Data shown as the mean ± s.d. of *n* =  3 replicates. d) Bioorthogonal reactions used to functionalize the AAV capsid at different ncAAs. e) Labeling AAV capsid with fluorescence dyes using different chemistries followed by SDS‐PAGE and fluorescence imaging confirms selective modification of VP1 (Full‐length SDS‐PAGE available in Figure S11).

We hypothesized that ncAA incorporation could be better tolerated when targeted to only the minor capsid proteins VP1 or VP2 (approximately 5 copies per capsid). This approach is also practically meaningful, given our recent demonstration that extensive chemical modification of the capsid has detrimental effects on its infectivity, and optimal properties of AAV conjugates are achieved with 5–10 modifications per capsid.^[^ ^41^, ^45^ ^]^ Our recently established “split‐Cap” system, which genetically separates expression of the three capsid proteins from a single open reading frame, enables selective ncAA incorporation into minor capsid proteins (Figure 2a).^[^ ^41^, ^45^ ^]^ This system decouples the expression of a selected minor capsid gene from the AAV Cap gene by deleting its start codon, and reintroduces it individually *in trans* under a CMV promoter.

Using the split‐Cap system, we attempted to incorporate LCA, pAAF, CpK, and 5HTP into the T454 site of VP1 alone. In all four cases, virus production was observed at levels comparable to WT‐AAV2, regardless of ncAA presence (Figure 2b). This is expected, since AAV genomes can be packaged efficiently by VP3 alone, even in the absence of VP1.^[^ ^41^, ^45^ ^]^ However, the resulting capsids lacking VP1 should be non‐infective, since VP1 is essential for endosomal escape. Indeed, viruses produced in the presence of each ncAA showed robust infectivity when tested on HEK293T cells at a constant MOI of 50 (Figures 2c, S2), but not the corresponding controls without ncAAs. The comparable titer and infectivity of VP1‐ncAA viruses relative to WT‐AAV2 (Figure 2b and c) indicate that ncAA incorporation into VP1 is tolerated well.

Each of the four ncAAs used above contains a bioorthogonal conjugation handle. Next, we sought to demonstrate the use of these handles for chemoselective labeling of the AAV capsid using various conjugation chemistries (Figure 2d). Viruses containing azide handles at VP1 (LCA and pAAF) were labeled with DBCO‐TAMRA (Figure S3) through strain‐promoted azide‐alkyne cycloaddition (SPAAC),^[^ ^64^ ^]^ VP1‐CpK virus was labeled using tetrazine‐fluorescein (Figure S3) via IEDDA,^[^ ^52^, ^58^ ^]^ while VP1‐5HTP was subjected to our recently reported electrochemical conjugation strategy eCLIC using a tertiary aniline,^[^ ^65^ ^]^ followed by Cu(I)‐click reaction with rhodamine azide (Figure S3). SDS‐PAGE analysis followed by fluorescence imaging in each case showed robust labeling of VP1 but not VP2 or VP3, confirming successful incorporation and chemoselective labeling of the ncAA residues at VP1 (Figure 2e).

Each of the aaRS/tRNA pairs used above is polyspecific in nature, i.e., these can incorporate several different structurally similar ncAAs while discriminating against the canonical amino acids. This property offers the opportunity to incorporate many additional chemistries into the AAV capsid using the same packaging machinery. We demonstrated this advantage using the polyspecific EcLeuRS/tRNA pair.^[^ ^55^, ^56^ ^]^ The same packaging system was used to incorporate nine different ncAAs into AAV2‐VP1‐454‐TAG (Figure S4a), containing diverse chemical handles such as ketone, alkyne, azide, strained alkene, and diazirine. In each case, the virus titer was comparable to WT‐AAV2 (Figure S4b). Furthermore, the robust infectivity of the resulting virus in each case – relative to a no‐ncAA control (lacks VP1) – confirmed successful ncAA incorporation (Figure S4c, d). These advances significantly expand the ncAA toolbox that can be introduced into the AAV capsid, and the chemistries that can be used for subsequent capsid engineering.

### AAV2‐Nanobody Conjugation Enabled by a Tetrazine‐ncAA

We recently demonstrated efficient retargeting of AAV to specific cell‐surface receptors by chemically conjugating a suitable antibody or nanobody to the capsid.^[^ ^45^ ^]^ The ability to chemically attach recombinant proteins to the AAV capsid in this manner opens up numerous exciting ways to engineer capsid properties. However, the SPAAC chemistry used to modify the AzK residue could not facilitate efficient conjugation between the AAV capsid and recombinant proteins.^[^ ^45^ ^]^ This is due to a combination of A) relatively slow kinetics of the SPAAC reaction, B) further retardation of reaction kinetics associated with the conjugation of two large molecules (AAV and a protein), and C) practical limits on the concentrations of AAV and protein that can be used to overcome the slow reaction rates. Instead, the inverse‐electron demand Diels–Alder reaction (IEDDA) between tetrazine and *trans*‐cyclooctene (TCO), which is orders of magnitude faster than SPAAC, enabled efficient AAV‐protein conjugation.^[^ ^45^ ^]^ However, since we lacked the ability to incorporate tetrazine‐ or TCO‐ncAAs into the AAV capsid at that time, we had to indirectly introduce them onto AzK‐labeled AAV using bifunctional DBCO‐tetrazine/TCO reagents. The ability to directly incorporate tetrazine/TCO handles would simplify this approach and enable facile attachment of macromolecules onto AAV capsids.

**Figure 3:**
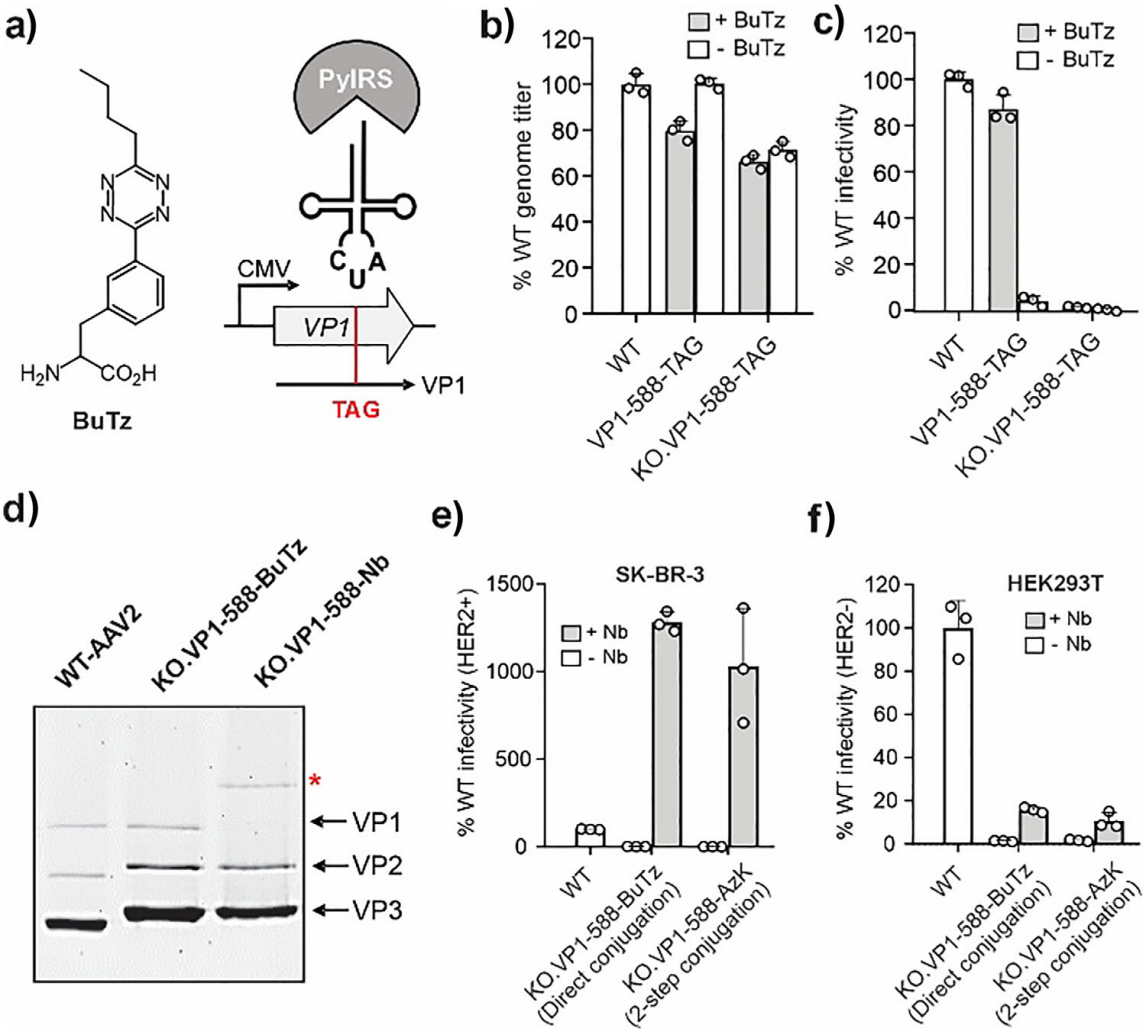
a) Structure of BuTz. genomic titer (b) and infectivity c) of AAV2 (WT and KO), containing BuTz at site 588 in VP1, in the presence or absence of 0.2 mM ncAA in the media. Packaged genome titer was measured by qPCR and was normalized to WT AAV2 titer. Infectivity was measured using the characteristic fluorescence from the expression of an encoded EGFP reporter, upon infecting HEK293T cells at a constant MOI of 50, and was normalized to WT AAV2. d) SDS‐PAGE analysis of the conjugation reaction between KO.VP1‐588‐BuTz and DBCO‐PEG12‐TCO‐functionalized Nb‐69‐AzK conjugation. The asterisk highlights the band corresponding to the VP1‐Nb conjugate (Figure S11 shows full image). e) Infectivity of WT, KO.VP1‐588‐BuTz, and KO.VP1‐588Nb, measured by the luminescence from an encoded luciferase reporter, upon infecting SK‐BR‐3 cells (high HER2 expression) at a constant MOI of 125, and was normalized to WT AAV2. f) Infectivity of WT, KO.VP1‐588‐BuTz, and KO.VP1‐588Nb, measured as above, upon infecting HEK293T cells (low HER2 expression) at a constant MOI of 50, and was normalized to WT AAV2 (mean ± s.d. *n* = 3). Comparison to a previously reported AAV2‐Nb conjugate,^[^ ^45^ ^]^ generated by 2‐step labeling of KO.VP1‐588‐AzK, is also shown in both (e) and f).

Using the engineered pyrrolysyl pair developed by the Mehl lab,^[^ ^54^ ^]^ we aimed to incorporate the tetrazine‐ncAA Tet‐v3.0‐Bu (BuTz) selectively at site R588 of VP1 (Figure 3a); conjugation of retargeting ligands onto the R588 site afforded the highest retargeting efficiency in the past.^[^ ^45^ ^]^ We observed successful production of VP1‐BuTz mutant virus at approximately 70% of wild‐type AAV2 titer in the presence of the ncAA (Figure 3b). As before, only the virus packaged in the presence of the ncAA was infective (Figures 3c, S5), and the infectivity was comparable to wild‐type AAV2.

We have previously found that retargeting AAV2 to a new receptor through the chemical attachment of novel receptor‐binding ligands works best when the virus is first “detargeted” from its native primary receptor, heparan sulfate proteoglycan (HSPG).^[^ ^41^, ^44^, ^45^ ^]^ This can be readily achieved by mutating conserved HSPG‐binding residues R585 and R588 to Ala across all 60 capsid proteins. To pursue such retargeting applications, we incorporated BuTz into VP1‐588‐TAG of a detargeted “KO” AAV2 capsid, containing R585A/R588A mutations. The resulting virus, KO.VP1‐588‐BuTz, was produced efficiently and was non‐infective, as expected (Figures 3b,c, S5). Next, we tested the feasibility of directly attaching anti‐HER2 nanobody 5F7 onto KO.VP1‐588‐BuTz capsids, and whether it retargets the virus to HER2‐expressing cells. To this end, AzK was incorporated into site 69 of 5F7, and the resulting protein was functionalized with TCO using a DBCO‐PEG12‐TCO reagent, as described previously (Figure S6).^[^ ^45^ ^]^ The TCO‐containing 5F7 was then added to KO.VP1‐588‐BuTz capsids at 1 µM final concentration. SDS‐PAGE showed efficient attachment of the nanobody onto VP1‐BuTz (Figure 3d). The resulting AAV2‐nanobody conjugate (KO.VP1‐588‐Nb) showed >12‐fold greater infectivity on HER2+ SK‐BR‐3 cells compared to its WT counterpart (Figure 3e), while showing >5‐fold lower infectivity on HEK293T cells that lack HER2 overexpression (Figure 3f). The retargeting efficiency of this direct AAV‐Nb conjugate was comparable to our previously reported counterpart generated in 2 steps from KO.VP1‐588‐AzK.^[^ ^45^ ^]^

### Optimized PEGylation Reduces AAV2 Immunogenicity In vivo

Adaptive immune response against AAV vectors is a significant challenge that precludes repeat‐dosing, significantly limiting the scope of such gene therapies. Chemical attachment of polymers such as polyethylene glycol (PEG) has been attempted to reduce the immunogenicity of AAV, but previous approaches lacked the precise control over site and stoichiometry of capsid modification that our platform offers.^[^ ^4^, ^66^, ^67^, ^68^ ^]^ We sought to explore how systematically varying the location and number of attachment sites per capsid affects PEGylation outcomes. We selected SPAAC chemistry to introduce PEG groups on AAV because: (A) unlike recombinant proteins, PEG reagents can be used at much higher concentrations to overcome the slower kinetics of SPAAC and achieve efficient labeling, and (B) PEG reagents for SPAAC chemistry are more readily available from commercial sources.

**Figure 4:**
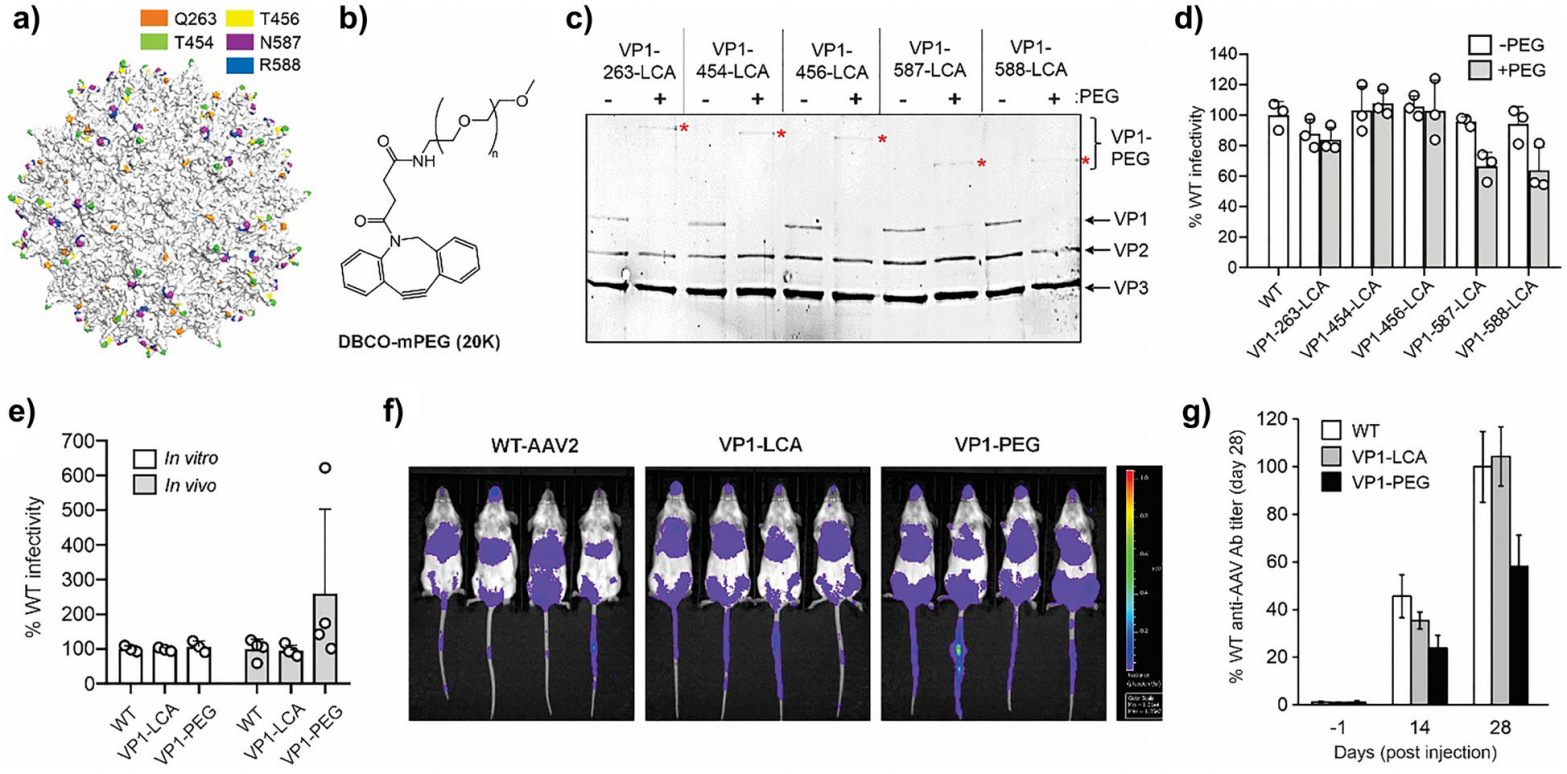
a) Distribution of sites targeted for LCA incorporation into AAV2. b) Structure of DBCO‐mPEG (MW 20 K Da). c) SDS‐PAGE analysis of the PEGylation reaction of AAV2‐VP1‐LCA mutants (Figure S11 shows full gel). d) Infectivity of AAV2‐VP1‐LCA mutants before and after PEGylation, normalized to the percentage infectivity of WT AAV2, measured by the expression of an encoded EGFP reporter, upon infecting HEK293T cells at a constant MOI 50 (see Figure S9). (mean ± s.d. of *n* = 3 replicates). e) In vitro and in vivo infectivity of WT‐AAV2, VP1‐LCA (site 454) mutant and its PEGylated counterpart (VP1‐PEG), normalized to WT‐AAV2 infectivity in each case. In vitro infectivity was measured using the expression of the luciferase reporter upon infecting HEK293T cells at a constant MOI 50 (mean ± s.d. *n* =  3). In vivo infectivity was evaluated at 4 weeks after injecting mice with 5x10^10 gc each, by measuring total flux from whole‐body luminescence imaging (mean ± s.d. *n* = 4). f) In vivo luminescence imaging (ventral) of mice at 4 weeks after vector injection. g) Anti‐AAV antibody levels in serum isolated from mice injected with different vectors at different time points, measured by ELISA and normalized to antibody levels observed for WT‐AAV2 on day 28 (mean ± s.d. *n* = 4).

To this end, we first used the EcLeuRS/tRNA pair to incorporate the azide‐containing amino acid LCA (Figure S7a) at 5 different highly surface‐exposed sites on VP1: T454, T456, N587, R588, and Q263 (Figure 4a). Each mutant was produced with wild‐type‐like titer and infectivity, and the latter was contingent upon the presence of ncAA in culture medium (Figure S7c,d, S8). Treatment of the resulting capsids with DBCO‐mPEG (Figure 4b; MW 20000) resulted in near‐complete conversion of VP1 to a higher molecular weight band in each case (Figure 4c), indicating efficient labeling. PEGylation had a modest impact on the infectivity of the 587 and 588 mutants, but not on the other three mutants (Figure 4d, S9). These observations suggest that PEGylation of VP1 alone is generally well‐tolerated by AAV.

Next, we evaluated the impact of capsid PEGylation on AAV immunogenicity in vivo. 5.0x10^10^ genome copies each of WT, VP1‐LCA (site 454), or PEGylated VP1‐LCA (VP1‐PEG) were intravascularly delivered to mice by tail‐vein injection (four adult female mice per group). Imaging the animals for the expression of the vector‐encoded luciferase reporter four weeks post‐injection revealed slightly elevated infectivity of the PEGylated virus relative to the other two (Figure 4e and f), even though all three virus preparations had comparable infectivity in vitro (Figure 4e), possibly due to slower clearance in vivo. ELISA analysis of serum harvested from the animals showed comparable anti‐AAV antibody levels for WT and VP1‐LCA mutant, but significantly lower levels for the PEGylated capsids (Figure 4g). These experiments demonstrate that VP1‐only PEGylation at permissive sites reduces immunogenicity of AAV2 without compromising infectivity. Future exploration of additional immunomodulatory attachments using our strategy holds potential to attenuate AAV immunogenicity even further.

### Simultaneous Incorporation of Two Distinct ncAAs Into AAV Capsid

Until now, GCE technology for AAV has been restricted to incorporating a single ncAA, AzK, using the pyrrolysyl pair. The ability to simultaneously incorporate multiple distinct ncAAs into the AAV capsid site‐specifically would unlock numerous next‐generation applications to probe and engineer this vector.^[^ ^36^, ^55^, ^63^, ^69^ ^]^ Such dual incorporation would need two engineered aaRS/tRNA pairs that can each read a distinct nonsense codon without cross‐reacting with each other. Having successfully demonstrated that we can now incorporate diverse ncAAs using four different aaRS/tRNA pairs into the AAV capsid, many of which exhibit mutual orthogonality, this ambitious goal is now within reach.

**Figure 5:**
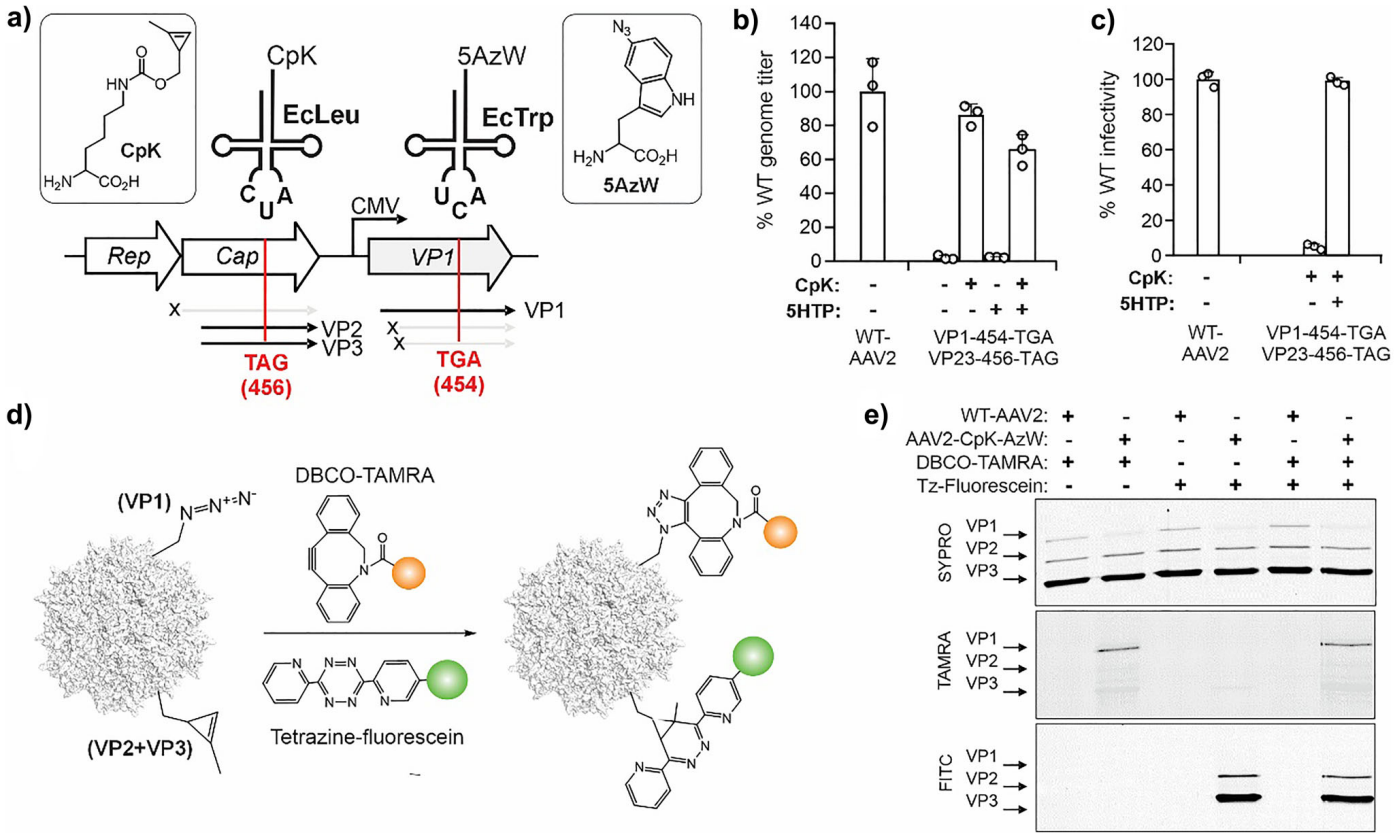
a) Scheme for packaging AAV2 incorporating two distinct ncAAs at different capsid proteins. b) Production of WT AAV2 and the dual‐ncAA mutants (packaged genome copies measured by qPCR) in the presence or absence of ncAAs in the media, normalized to the percentage of WT AAV2 titer. c) Infectivity of these viruses, measured by the expression of an encoded EGFP reporter, upon infecting HEK293T cells at a constant MOI 50 (mean ± s.d. of *n* = 3). d) Reaction scheme for functionalizing CpK‐AzW‐AAV2 capsid using mutually compatible SPAAC and IEDDA chemistries. e) SDS‐PAGE and fluorescence imaging of these labeling reactions show selective labeling of VP2 + VP3 with tetrazine‐fluorescein and VP1 with DBCO‐TAMRA (Figure S11 shows full gel).

To establish proof‐of‐concept for such dual ncAA incorporation into AAV, we selected EcTrpRS/tRNA_UCA_ and EcLeuRS/tRNA_CUA_ for incorporating 5AzW and CpK, in response to TGA and TAG, respectively (Figure 5a). These pairs are mutually orthogonal, and the ncAA combination introduces a pair of compatible bioorthogonal conjugation handles, which can be independently functionalized afterwards using SPAAC and IEDDA chemistries, respectively.^[^ ^63^ ^]^ In our split‐Cap expression system, we introduced the TAG codon in the ORF encoding VP2 + VP3 at the T456 site, while the VP1‐coding ORF was mutated to TGA at T454 (Figure 5a).

We constructed plasmids co‐expressing both aaRS/tRNA pairs alongside the components of the split‐Cap packaging system. Co‐transfecting these plasmids into HEK293T cells in the presence of both CpK and AzW led to robust AAV packaging, as measured by qPCR (Figure 5b). No virus packaging was observed when CpK was omitted from the growth medium, since the major capsid protein VP3 could not be expressed (Figure 5b). However, when CpK was present, robust virus titers were observed even in the absence of 5AzW (Figure 5b). This is because VP3 alone can package non‐infective capsids, even in the absence of VP1 expression. However, capsids produced in the absence of 5AzW were non‐infective, whereas the ones packaged in the presence of both ncAAs showed infectivity comparable to WT‐AAV2 (Figure 5c). Incorporation of 5AzW alone into VP1, using a VP1‐454‐TGA/WT‐VP23 construct, yielded similar results (Figure S10). To further confirm the presence of CpK in VP2/VP3, and 5AzW in VP1, we treated the dually‐labeled capsids with DBCO‐TAMRA, and tetrazine fluorescein (Figure 5d). As expected, treatment with the former resulted in selective TAMRA‐labeling of the VP1 band, while the latter only yielded fluorescein‐labeled VP2/3 bands, as observed by SDS‐PAGE followed by fluorescence imaging (Figure 5e). We also demonstrated dual capsid labeling with both fluorophores through sequential incubation with both reagents. These experiments for the first time demonstrate the feasibility of incorporating two distinct ncAAs into the AAV capsid, and their use for subsequent site‐specific bioorthogonal attachment of two distinct entities.

## Conclusion

The work presented here drastically expands the GCE toolbox for AAV engineering by overcoming the long‐standing limitation that largely restricted the field to a single ncAA, AzK. We demonstrate that the sensitivity of the AAV capsid to diverse ncAA substitutions can be circumvented by targeting incorporation selectively to minor capsid proteins rather than all 60 capsid proteins. This strategy enabled successful incorporation of numerous ncAAs with diverse chemical functionalities using four different aaRS/tRNA pairs, including azide, alkyne, strained alkene, ketone, hydroxyindole, tetrazine, and diazirine. We showcase the utility of these expanded capabilities through multiple proof‐of‐concept applications: direct tetrazine incorporation enabled ultrafast IEDDA‐mediated protein conjugation for efficient HER2‐targeted gene delivery, optimized PEGylation reduced immunogenicity while preserving infectivity, and dual ncAA incorporation demonstrated the feasibility of simultaneous, orthogonal capsid modifications. Our technology also facilitated the production of ncAA‐labeled AAV at WT‐like titer and infectivity, even in a challenging scenario such as dual ncAA incorporation.

These technological advances offer exciting opportunities for rational AAV engineering. The ability to incorporate diverse bioorthogonal handles directly into capsids will streamline conjugation protocols and enable attachment of challenging payloads, from complex proteins to synthetic polymers and drug molecules, with unprecedented versatility. Dual ncAA incorporation holds much potential, allowing, for example, simultaneous installation of targeting ligands and immunomodulatory agents, or enabling controlled release mechanisms through orthogonal chemistries. Beyond the specific applications demonstrated here, our expanded ncAA repertoire provides a foundation for engineering AAV vectors with tailored pharmacokinetics, enhanced tissue specificity, and reduced immunogenicity — addressing key challenges that have limited the clinical translation of AAV‐based gene therapies.

## Conflict of Interests

A patent application has been submitted based on the technology described herein, where AC and QP are co‐inventors. AC is a cofounder and senior advisor at BrickBio, Inc.

## Supporting information



Supporting Information

## Data Availability

The data that support the findings of this study are available from the corresponding author upon reasonable request.
