# Supplementary material for: An Expanded Toolbox for Versatile Chemical Editing of Adeno‐Associated Virus
Source: Angew Chem Int Ed Engl. 2026 Jan 1;65(7):e16157. doi: 10.1002/anie.202516157 (PMC12887607; doi:10.1002/anie.202516157)
Supplement: Supplementary file 1 — Supporting Information [file ANIE-65-e16157-s001.pdf]

Supporting information for:

An Expanded Toolbox for Versatile Chemical Editing of Adeno-Associated Virus

Quan Pham, Jake Glicksman, Boyang Han, David Koo, Conor Loynd, Soumya Jyoti Singha Roy, and Abhishek Chatterjee*

Department of Chemistry, Boston College, 2609 Beacon Street, Chestnut Hill, MA 02467, USA

**Corresponding author.* E-mail address: abhishek.chatterjee@bc.edu

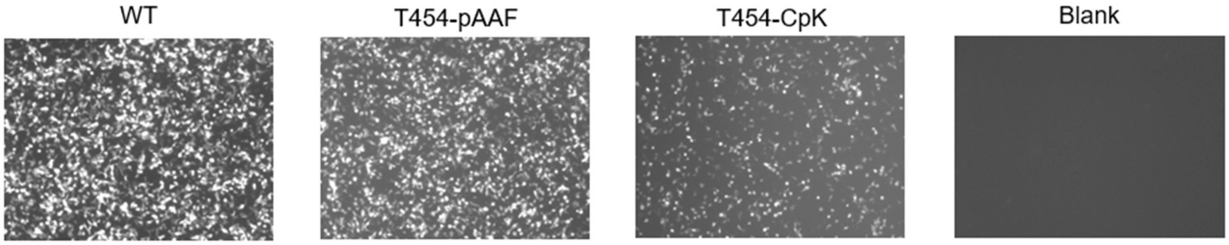

Figure S1. Fluorescence images of cells associated with the experiment described in Figure 1f. HEK293T cells were infected with a constant MOI (50) of wild-type or pAAF and CpK mutants of AAV2 at site T454, and imaged 48 h post-infection.

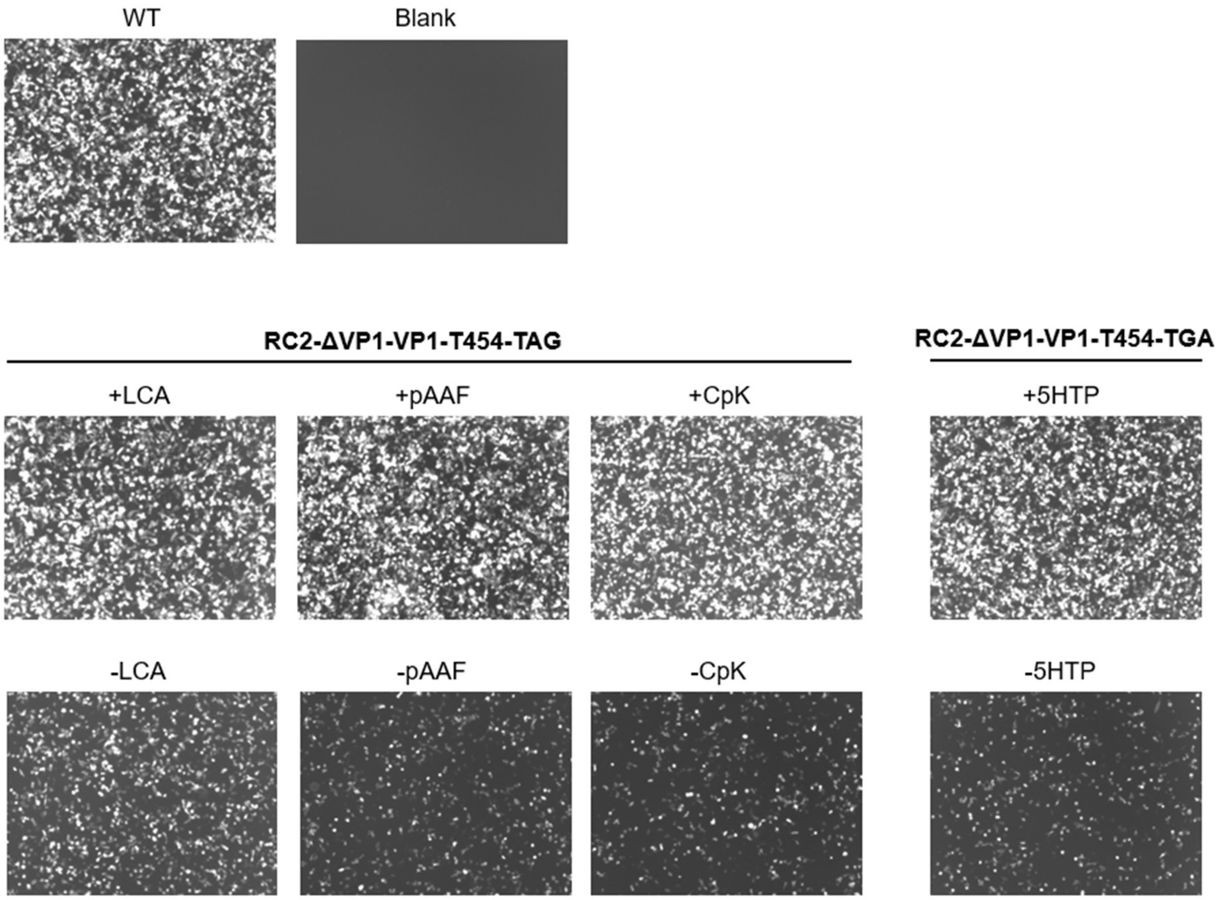

Figure S2. Fluorescence images of cells associated with the experiment described in Figure 2c. HEK293T cells were infected with a constant MOI (50) of wild-type or various ncAA mutants of AAV2 at site T454 of VP1, and imaged 48 h post-infection.

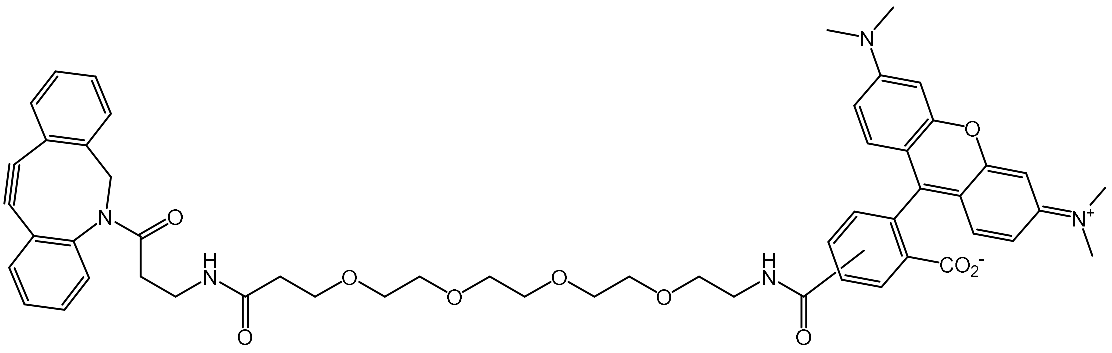

DBCO-TAMRA

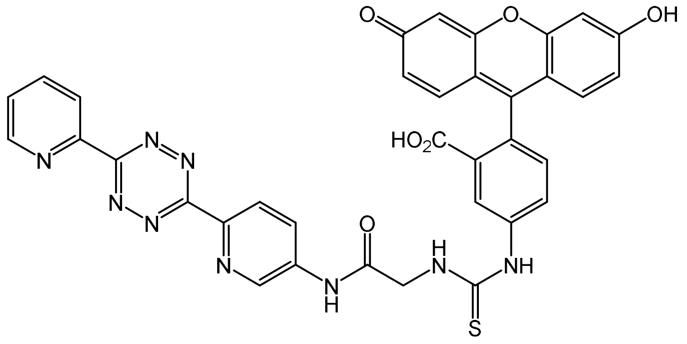

Tetrazine-Fluorescein

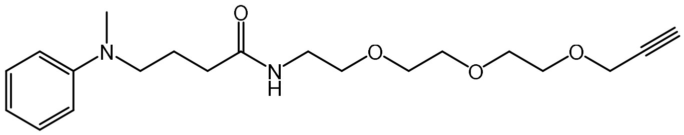

Aniline-alkyne

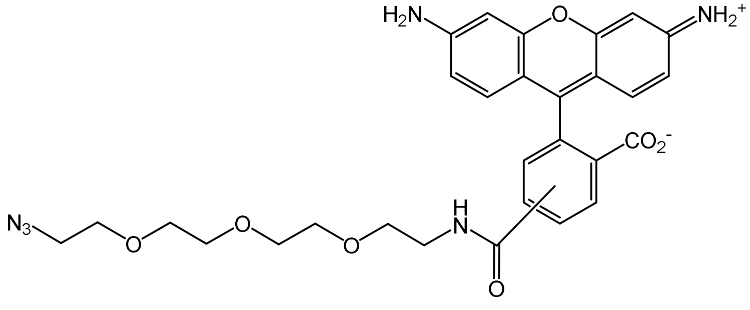

Azide-Rhodamine

Figure S3: Structures of labeling reagents used in experiments described in Figure 2d-e

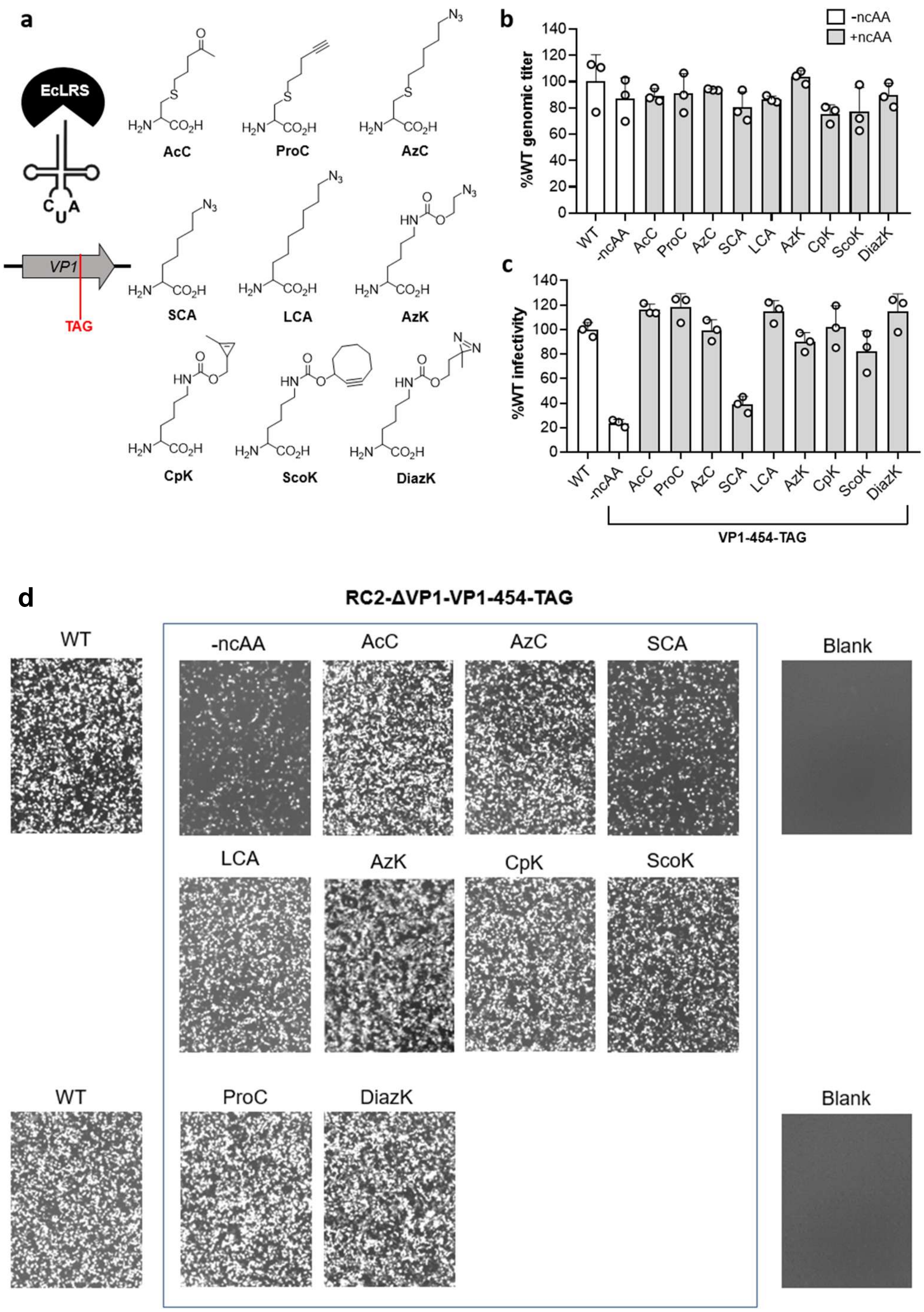

Figure S4: a) Chemical structures of various ncAAs tested for the incorporation in AAV2 capsid by the polyspecific EcLeu pair. b) Incorporation of various ncAAs into AAV2-VP1-454 (packaged genome copies measured by qPCR) in the presence or absence of ncAA in the media, normalized to the % of WT AAV2 titer. c) Infectivity of the resulting ncAA-mutants, normalized to WT AAV2, measured by the expression of an encoded EGFP reporter, upon infecting HEK293T cells at a constant MOI 50. (mean ± s.d. of $n = 3$). d) Fluorescence images of cells associated with the experiment described in panel c (48 h post-infection).

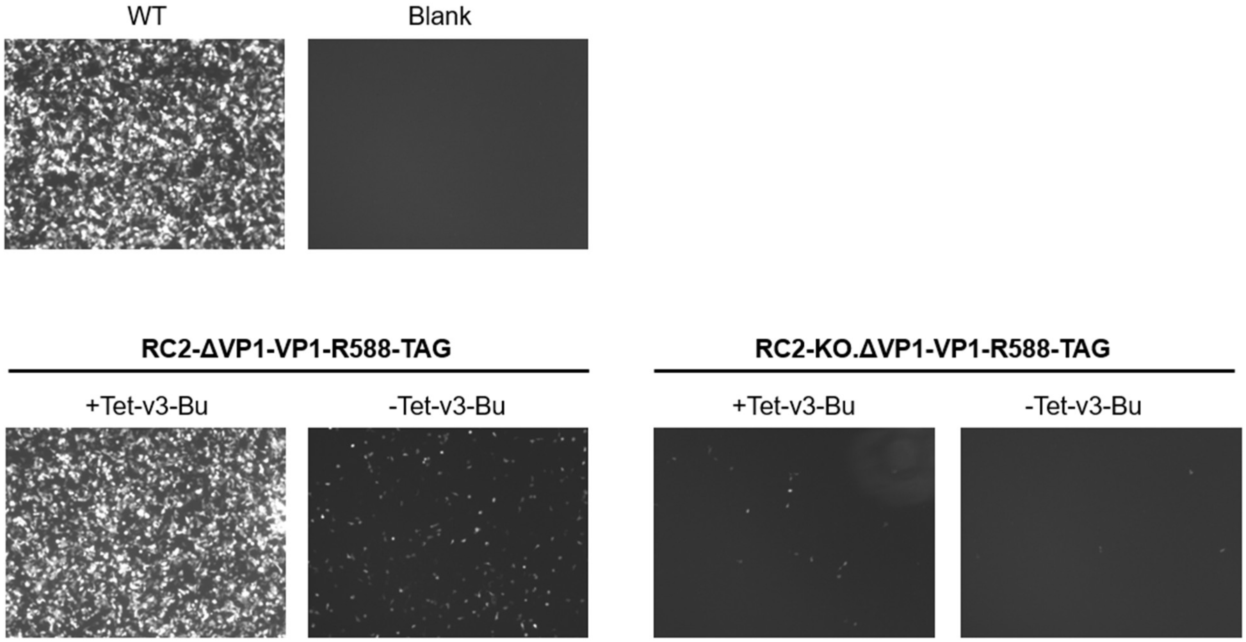

Figure S5. Fluorescence images of cells associated with the experiment described in Figure 3c. HEK293T cells were infected with a constant MOI (50) of WT AAV2 or VP1-588-BuTz mutants without or with the R585A/R588A mutations (KO) for the virus from the native HSPG receptor, and imaged 48 h post-infection.

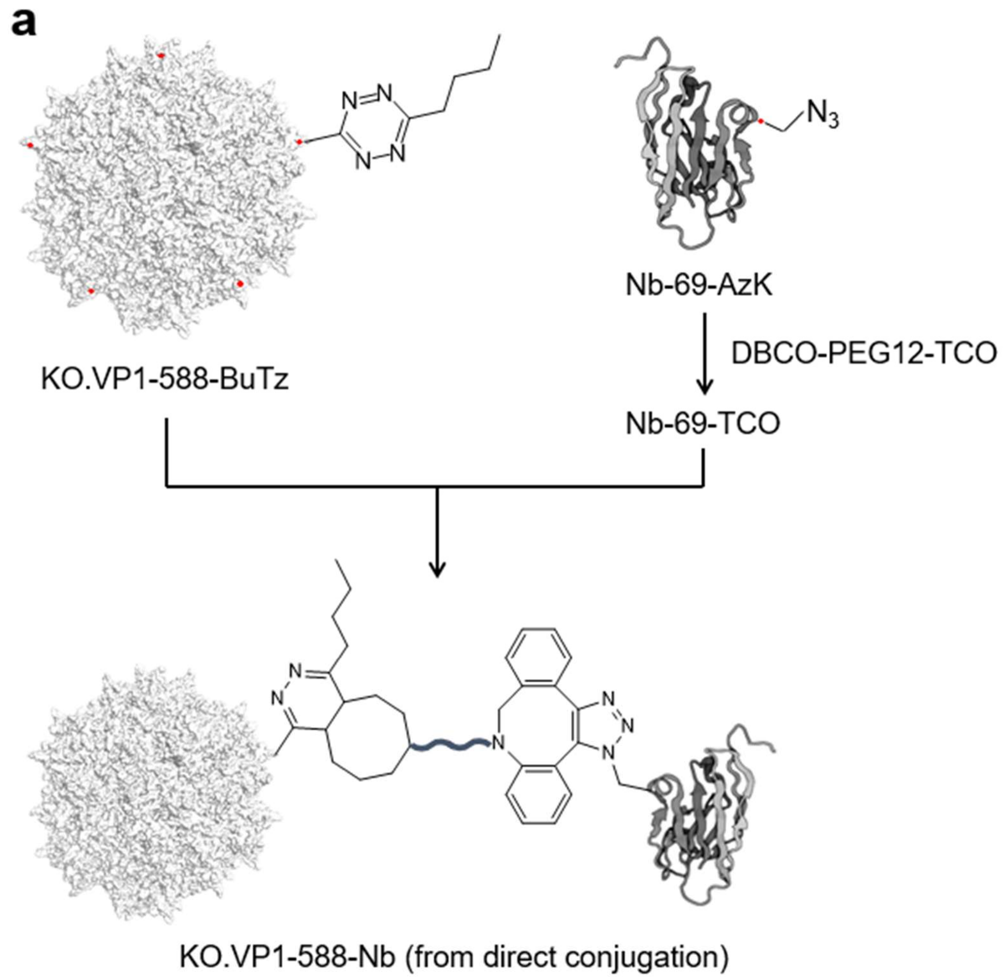

a
KO.VP1-588-BuTz
Nb-69-AzK
DBCO-PEG12-TCO
Nb-69-TCO
KO.VP1-588-Nb (from direct conjugation)

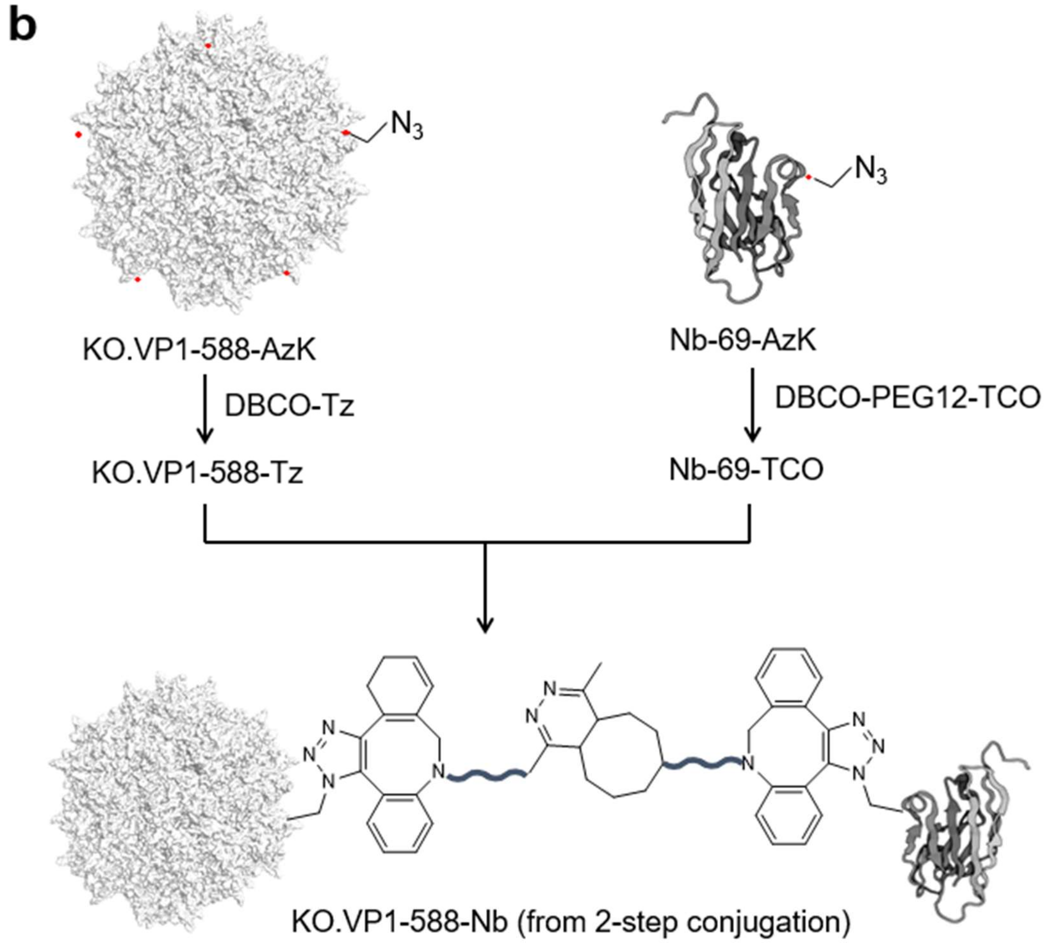

Figure S6: a) Scheme for direct chemically conjugating the 5F7 nanobody to the AAV-BuTz capsid using the IEDDA chemistry. b) Scheme for 2-step chemically conjugating the 5F7 nanobody to the AAV-AzK capsid using the SPAAC and IEDDA chemistry.

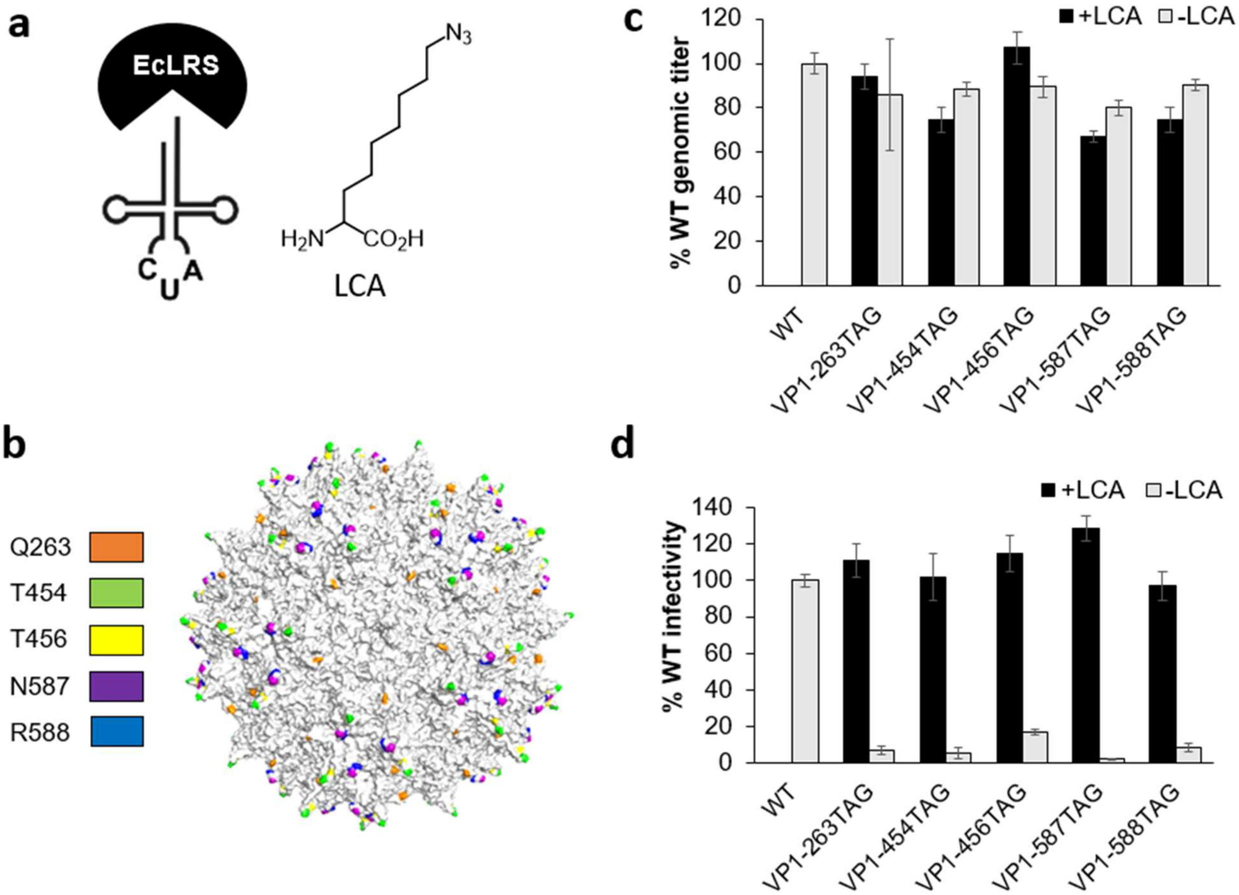

Figure S7: a) Structure of LCA which can be incorporated using the engineered EcLeu pair. b) A color-coded depiction of the distribution of sites targeted for LCA incorporation in the AAV2 capsid selectively in VP1. c) Production of LCA-mutants of AAV2 at various sites of VP1 protein (packaged genome copies measured by qPCR) in the presence or absence of ncAA in the media, normalized to the % of WT AAV2 titer. d) Infectivity of AAV2 at various sites made in the presence or absence of ncAA in the media, measured by the expression of an encoded EGFP reporter, upon infecting HEK293T cells at a constant MOI 50, normalized to the percentage of WT AAV2 infectivity.

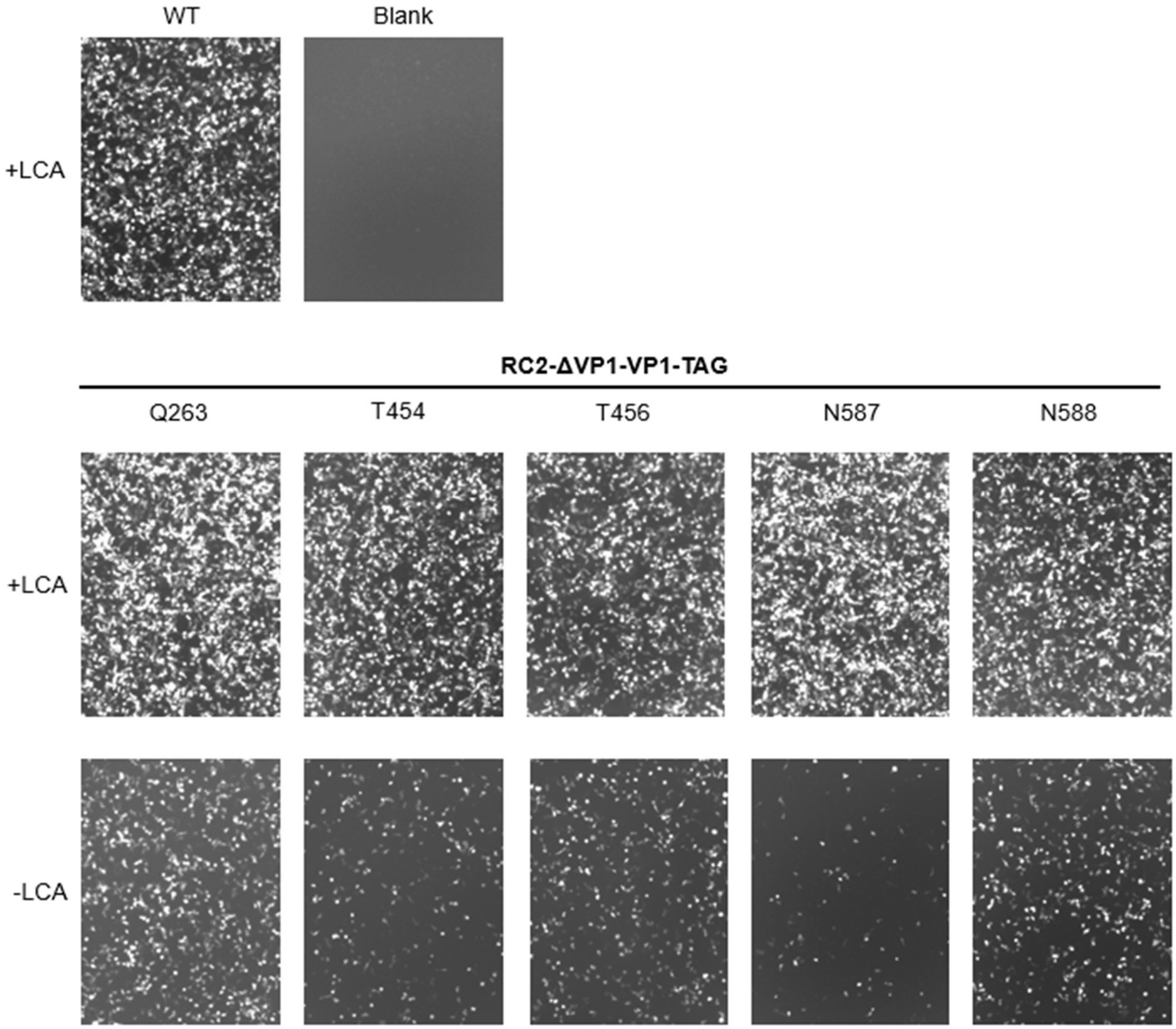

Figure S8: Fluorescence images of cells associated with the experiment described in Figure S7d. HEK293T cells were infected with a constant MOI (50) of wild-type or LCA-mutants of AAV2 at various sites of VP1 protein made in the presence or absence of ncAA in the growth media, and imaged 48 h post-infection.

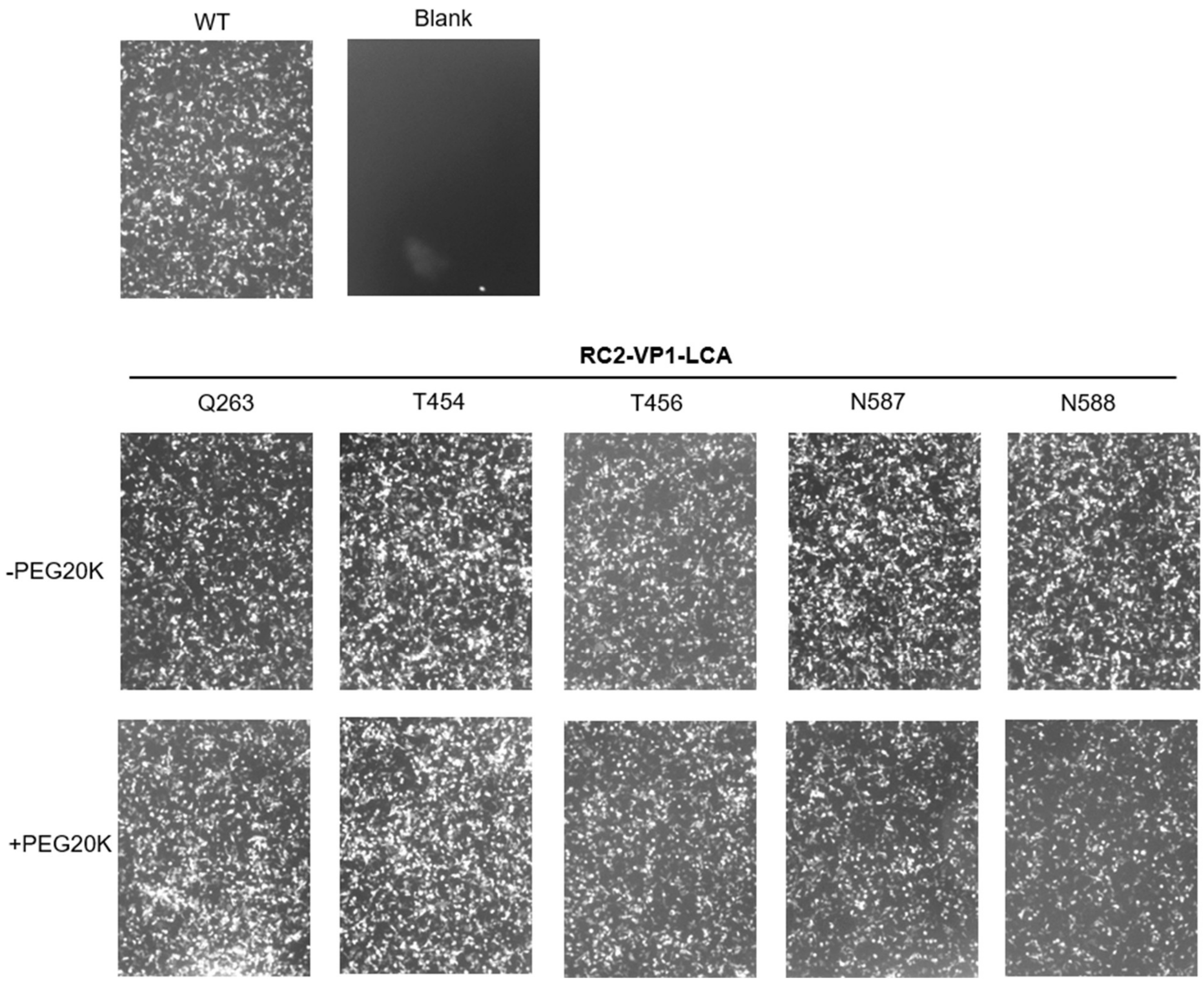

Figure S9: Fluorescence images of cells associated with the experiment described in Figure 4d. HEK293T cells were infected with a constant MOI (50) of wild-type or LCA mutants of AAV2 at various sites of VP1 without and with PEGylation, and imaged 48 h post-infection.

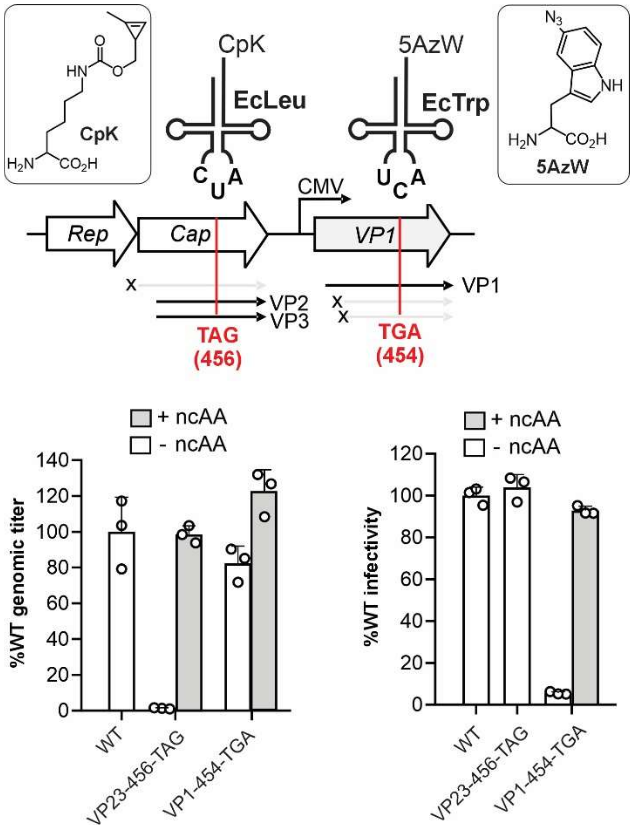

Figure S10: Control experiments evaluating the incorporation of a single-ncAA into the AAV capsid (only VP23-456-TAG, or only VP1-454-TGA) using the packaging system designed for dual-ncAA incorporation (Figure 5). A scheme describing the packaging system for incorporating two distinct ncAAs at different capsid proteins is shown on top. Production of WT AAV2 and the single-ncAA mutants (packaged genome copies measured by qPCR), and infectivity of these viruses, measured by the expression of an encoded EGFP reporter, upon infecting HEK293T cells at a constant MOI 50 (mean ± s.d. of $n = 3$), are shown below.

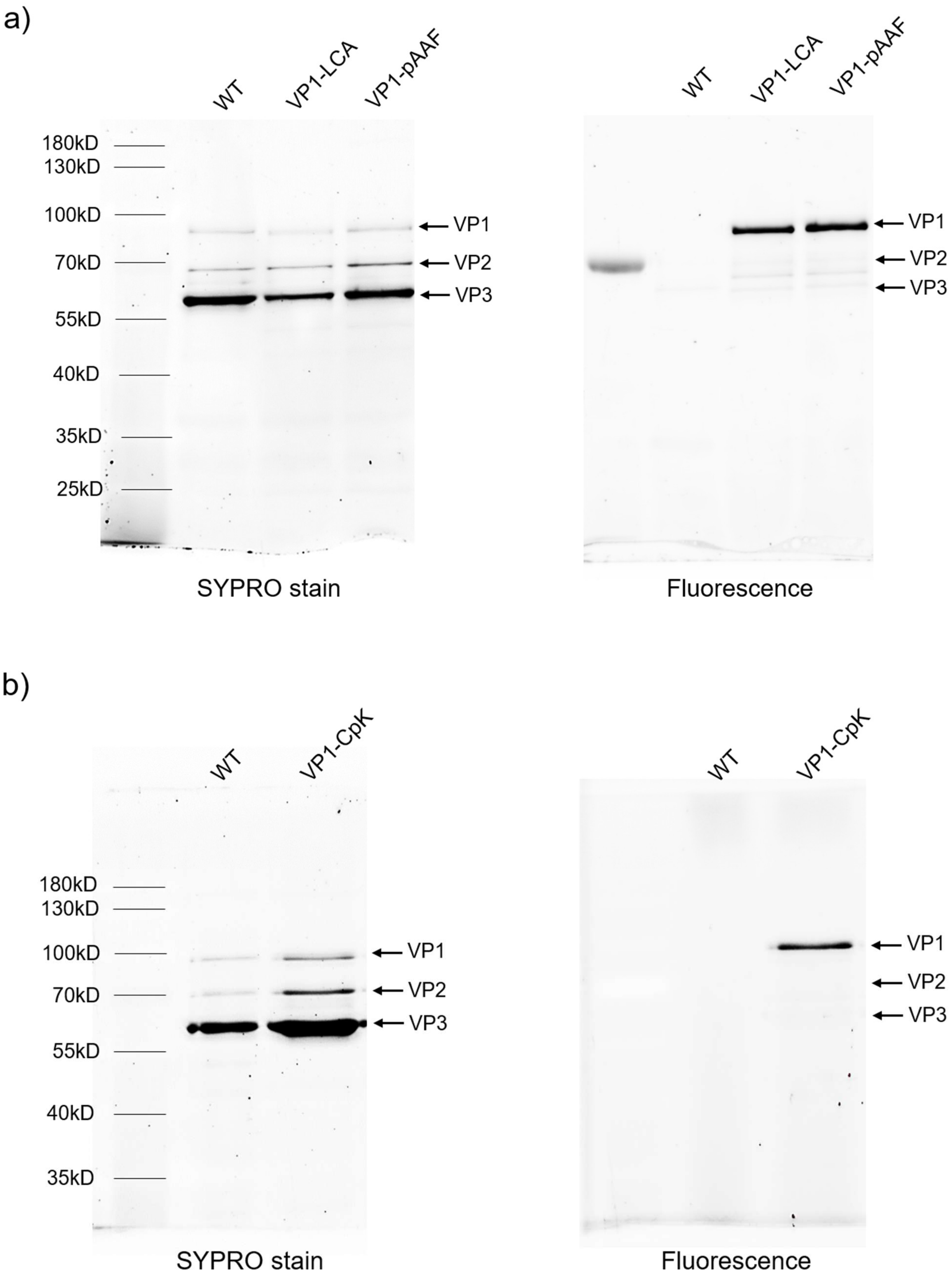
a)
WT
VP1-LCA
VP1-pAAF
180kD
130kD
100kD
70kD
55kD
40kD
35kD
25kD
VP1
VP2
VP3
SYPRO stain
WT
VP1-LCA
VP1-pAAF
VP1
VP2
VP3
Fluorescence
b)
WT
VP1-CpK
180kD
130kD
100kD
70kD
55kD
40kD
35kD
VP1
VP2
VP3
SYPRO stain
WT
VP1-CpK
VP1
VP2
VP3
Fluorescence

c)

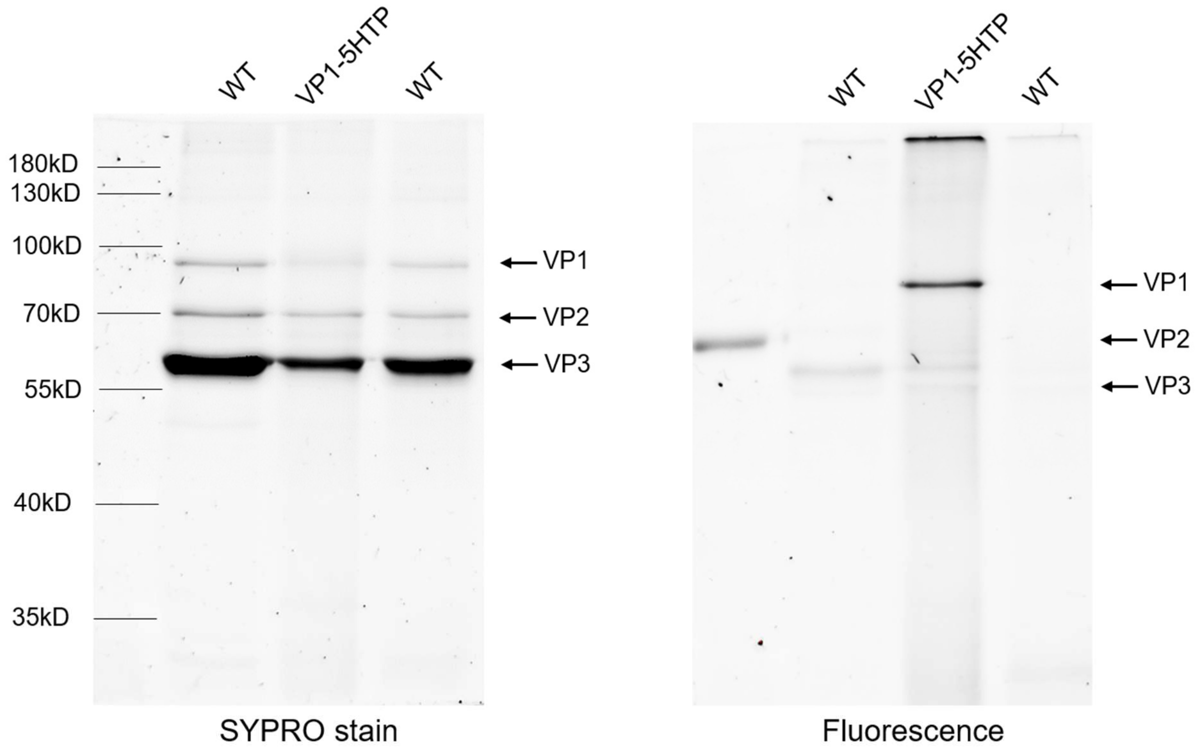
WT
VP1-5HTP
WT
180kD
130kD
100kD
70kD
55kD
40kD
35kD
VP1
VP2
VP3
SYPRO stain
WT
VP1-5HTP
WT
VP1
VP2
VP3
Fluorescence

d)

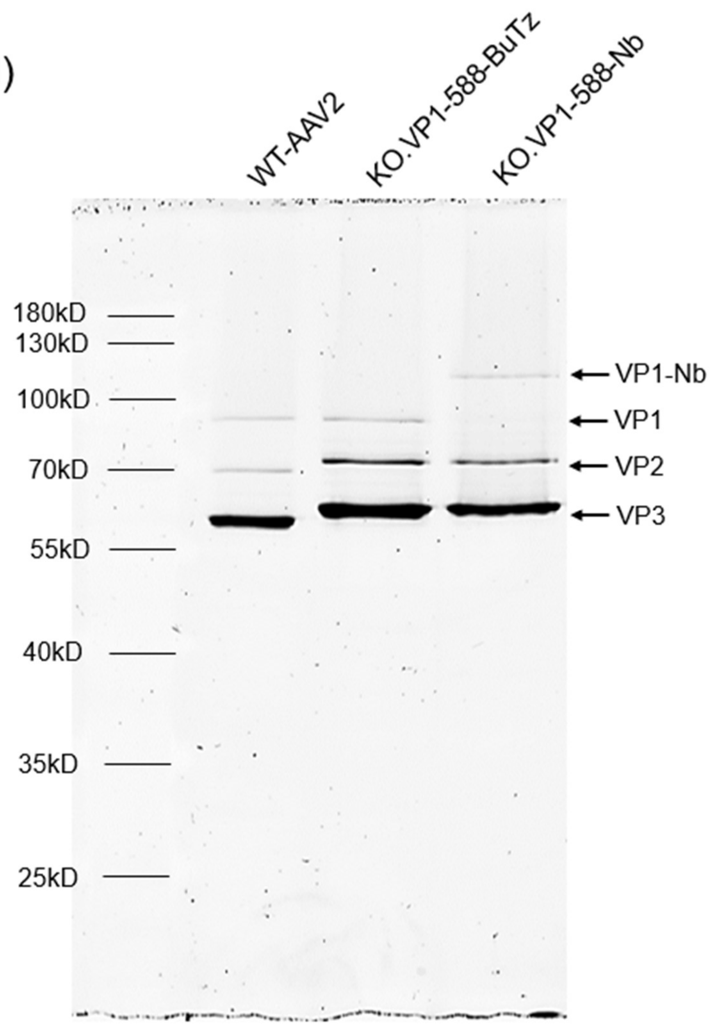
WT-AAV2
KO.VP1-588-BuTz
KO.VP1-588-Nb
180kD
130kD
100kD
70kD
55kD
40kD
35kD
25kD
VP1-Nb
VP1
VP2
VP3

e)

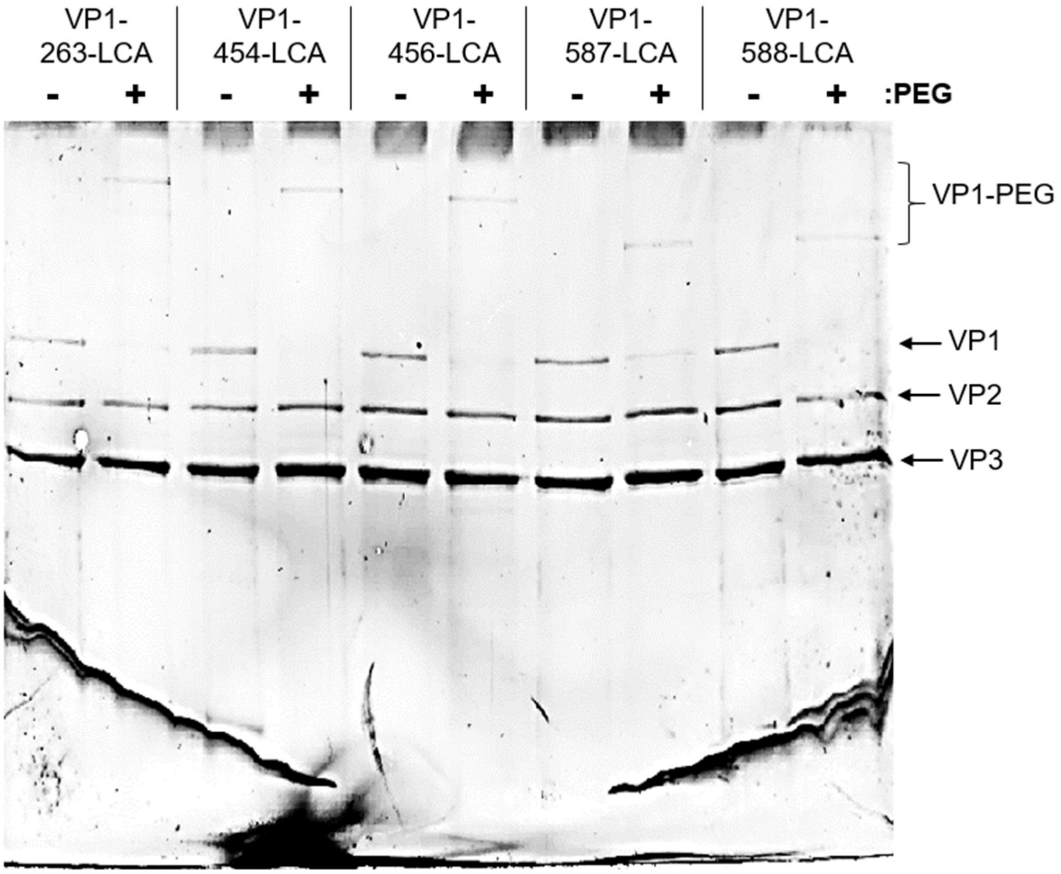
VP1-
263-LCA
VP1-
454-LCA
VP1-
456-LCA
VP1-
587-LCA
VP1-
588-LCA
- + - + - + - + - + :PEG
VP1-PEG
VP1
VP2
VP3

f)

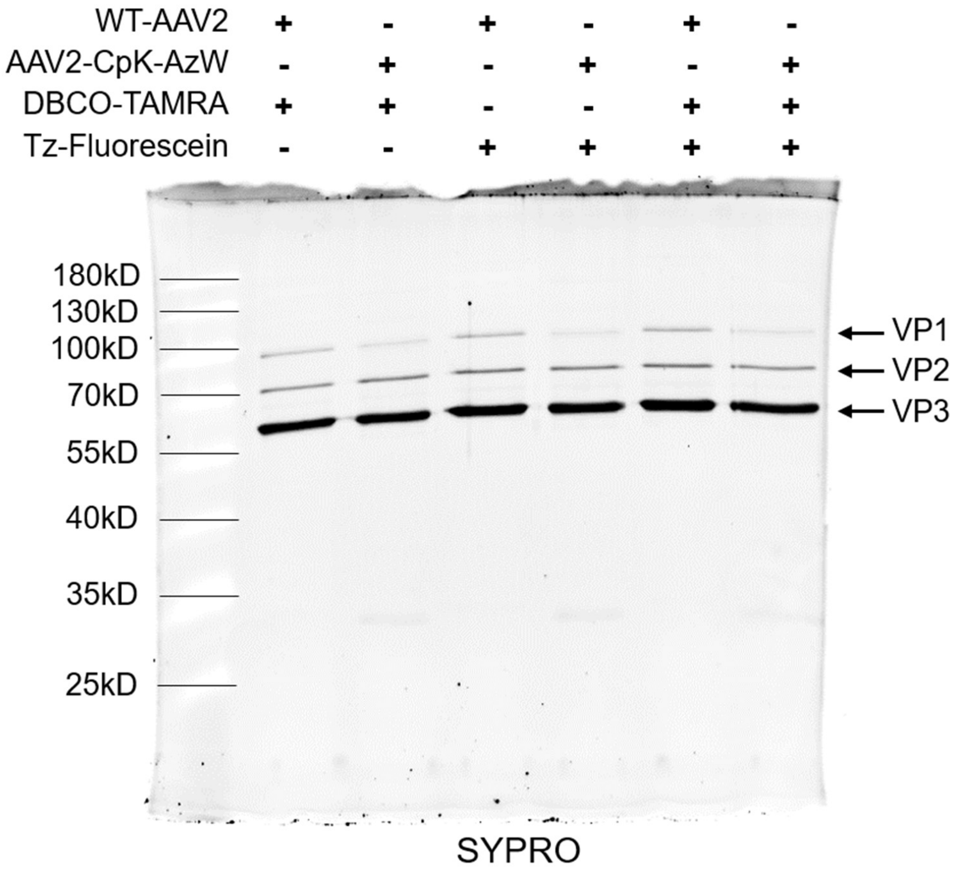
WT-AAV2 + - + - + -
AAV2-CpK-AzW - + - + - +
DBCO-TAMRA + + - - + +
Tz-Fluorescein - - + + + +
180kD
130kD
100kD
70kD
55kD
40kD
35kD
25kD
VP1
VP2
VP3
SYPRO

g)

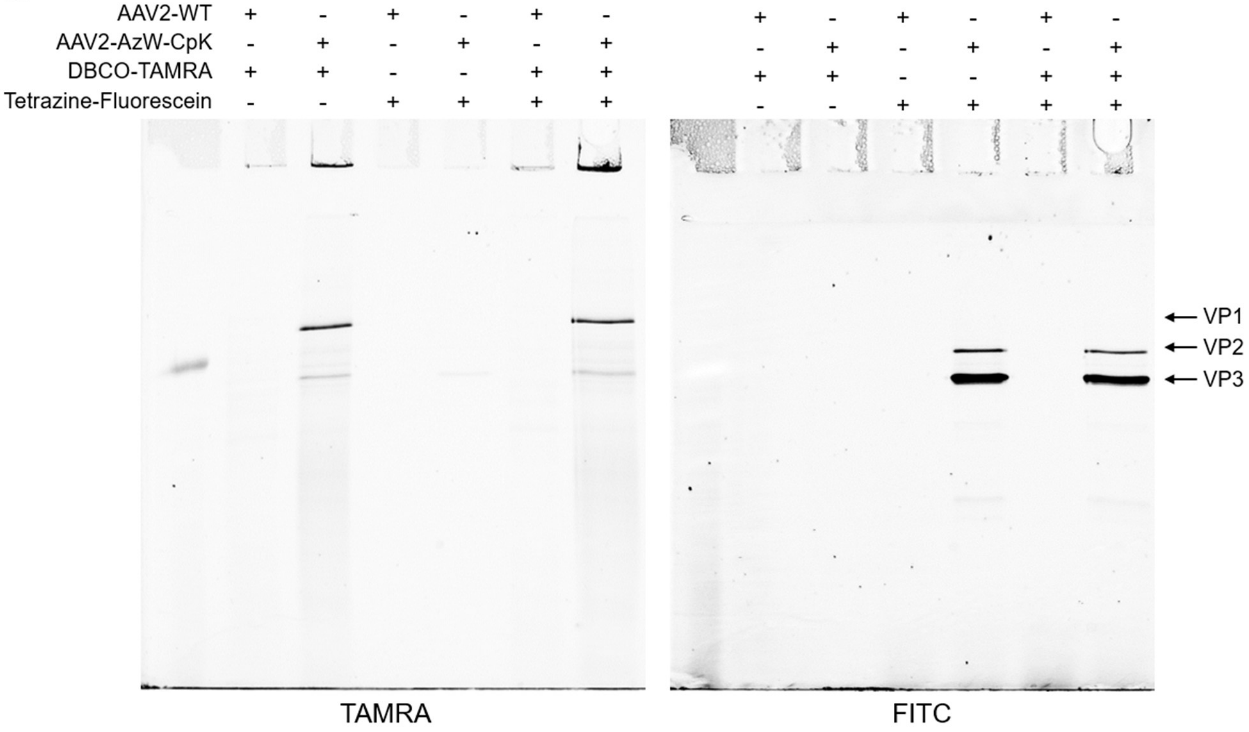

Figure S11: Full-length gel images

a) Figure 2e (Left)

b) Figure 2e (Middle)

c) Figure 2e (Right)

d) Figure 3d

e) Figure 4c

f) Figure 5e (Top)

g) Figure 5e (Middle and Bottom)

Materials and Methods

Cell Culture. HEK293T and SK-BR-3 cells were obtained and maintained as previously described.[1,2] Briefly, cell lines were cultured at 37 °C and 5% CO_2 in DMEM-high glucose (HyClone) media supplemented with penicillin/streptomycin (HyClone, final concentration of 100 U/mL penicillin and 100 µg/mL streptomycin) and 10% fetal bovine serum (Corning).

Non-canonical amino acids and fluorescein probes

Azido-lysine (AzK) was purchased from Iris Biotech GMBH (Germany). Diazirine-lysine (DiazK), strained cyclooctyne-L-lysine (SCOK), and Cyclopropene-L-lysine (CpK) were purchased from Sirius Fine Chemicals (Germany). Butyl Tetrazine-v3 (BuTz) was a gift from the Mehl lab (Oregon State University). 5-hydroxy-L-tryptophan (5-HTP) was purchased from Chem-Impex. Long-chain azide (LCA), short-chain azide (SCA), Azido-cysteine (AzC), propargyl-cysteine (PrC), acetyl-Cysteine (AcC), para-azido-amino-phenylalanine were chemically synthesized using previously established routes.[3]

DBCO-TAMRA and Rhodamine-azide were purchased from Click Chemistry Tools. Tetrazine-flourescein[3] and aniline-alkyne[4] were chemically synthesized using previously established routes.

Cloning and Plasmids.

Transformation was done in Top10 cells using a BioRad electroporator. DNA oligo synthesis and Sanger Sequencing were performed by Genewiz. Phusion polymerase was purchased from Thermo Scientific™, PrimeSTAR® Max DNA Polymerase was purchased from Takara, T4 DNA LIGASE was purchased from Qiagen.

pHelper[1], pIDTsmart-RC2-WT[5], pIDTsmart-RC2-454TAG[5], pIDTsmart-RC2-ΔVP1-VP1-TAG[5], pIDTsmart-RC2-ΔVP12-VP12-TAG[5], pIDTSmart-MbPylRS-4xPyltR-ITR-GFP[5] were described previously.

pIDTsmart-RC2-454-TGA cloning was done by overlap-extension PCR on pIDTsmart-RC2-WT using RC2-HindIII-F, RC2-454-TGA-F, RC2-454-TGA-R, Cap-SbfI-R primers. The PCR product was digested with HindIII and SbfI and cloned into pIDTsmart-RC2-WT with HindIII and SbfI sites. AAP's opal stop codon was mutated by PCR amplifying pIDTsmart-RC2-454-TGA with AAP-TGA-TAA-F and AAP-TGA-TAA-R primers, followed by Gibson assembly (Invitrogen™). Similarly, Rep's opal stop codon was mutated by PCR amplifying pIDTsmart-RC2-454-TGA with Rep-TGA-TAA-F and Rep-TGA-TAA-R primers, followed by Gibson assembly (Invitrogen™).

pIDTsmart-RC2- ΔVP1-CMV-VP1-454-TGA was generated from the original plasmid pIDTsmart-RC2-ΔVP1-VP1-WT. Overlap extension PCR was done with SbfI-CMV-F, RC2-454-TGA-F, RC2-454-TGA-R, Cap-Bsu36I-R. The PCR product was digested and cloned into pIDTsmart-RC2-delVP1-VP1-WT using SbfI and Bsu36I restriction sites. Rep's and AAP's opal stop codons were mutated to TAA by overlap-extension PCR on pIDTsmart-RC2-DelVP1 using RC2-HindIII-F, Cap-SbfI-R and suitable overlap primers. The PCR product was digested with HindIII and SbfI and cloned into pIDTsmart-RC2-DelVP1-CMV-VP1-454-TGA using HindIII and SbfI sites.

pIDTSmart-PLRS-4xLtR-ITR-GFP and pIDTSmart-PLRS-4xLtR-ITR-FLUC were generated from the original plasmid pIDTsmart-PLRS[3]. 4xLtR cassette was amplified by PrimeStar polymerase

on pIDTsmart-4xLtR using NheI-HTS25-F and SbfI-HTS25-R primers. The PCR product was digested with restriction enzymes NheI and SbfI and cloned into pIDTsmart- PolyLeuRS using SpeI and SbfI restriction sites, yielding the intermediate plasmid pIDTSmart- PolyLeuRS-4xLtR. Next, ITR-EFGP and ITR-FLUC were digested from the plasmid pIDTSmart-8xPyltR-ITR-GFP[1] and pIDTSmart-MbPylRS-4xPyltR-ITR-FLUC, respectively with restriction enzymes SbfI, and cloned into pIDTSmart-MbPylRS-4xPyOtR using SbfI restriction site.

pIDTSmart-YRS-4xYtR-ITR-GFP was generated from the original plasmid pIDTsmart-MbPylRS[1]. PCR was done on pB1U-EcYRS[6] with EcYRS-NheI-F and EcYRS-XhoI-R primers, digested and cloned into pIDTsmart-MbPylRS using NheI and XhoI restriction sites, yielding plasmid pIDTsmart-EcYRS. Next, the 4xYtR cassette was amplified by PrimeStar polymerase on pIDTsmart-4xYtR with NheI-HTS25-F and SbfI-HTS25-R primers. The PCR product was digested with restriction enzymes NheI and SbfI and cloned into pIDTsmart-EcYRS using SpeI and SbfI restriction sites, yielding plasmid pIDTSmart-EcYRS-4xYtR. Lastly, ITR-EFGP was digested from the plasmid pIDTSmart-8xPyltR-ITR-GFP[1] by SbfI enzyme and cloned into pIDTSmart-EcYRS-4xYtR using SbfI site.

pAAV-EcWRS-4xWtR-ITR-GFP was generated from the original plasmid pAAV-ITR-FLUC. The backbone vector was PCR amplified with NotI-ColE1-R and KpnI-F1ori-F primers. EcWRS gene was amplified from pIDTsmart-EcWRS[7] using NotI-SbfI-CMV-F and PolyA-KpnI-R primers and cloned into the amplified pAAV vector using NotI and KpnI restriction sites. Next, the 4xWtR cassette was amplified by PrimeStar polymerase on pIDTsmart-4xWtR with NheI-HTS25-F and SbfI-HTS25-R primers. The PCR product was digested with restriction enzymes NheI and SbfI, and cloned into pIDTsmart- EcYRS using SpeI and SbfI restriction sites, yielding plasmid pIDTSmart-EcWRS-4xWtR. Lastly, ITR-EFGP was digested from the plasmid pIDTSmart-8xPyltR-ITR-GFP by SbfI enzyme and cloned into pIDTSmart-EcWRS-4xWtR using SbfI site.

pIDTSmart-MbPylRS(R2-84)-4xPyltR-ITR-GFP was generated from the original plasmid pIDTsmart-MbPylRS-4xPyltR-ITR-GFP[2]. PCR was done on pIDTsmart-MbPylRS-4xPyltR-ITR-GFP with Mb-R284-F, 270G-R, 270G-F, 311G313A-R, 311G313A-F and Mb-R284-R respectively to introduce the R2-84 mutantion for the incorporation of BuTz[8]. The PCR products were purified and went through overlap PCR with Mb-R284-F and Mb-R284-R. The overlap PCR product was digested and cloned into pIDTsmart-MbPylRS-4xPyltR-ITR-GFP using NheI and SalI restriction sites, yielding plasmid pIDTSmart-MbPylRS(R2-84)-4xPyltR-ITR-GFP.

Primers used in this study:

Primer name	Sequence
RC2-HindIII-F	CGTCAGACGCGGAAGCTTCGATCAAC
RC2-454-TGA-F	CAAACACTCCAAGTGGATGAACCACGCAGTCAAGGCTTCAGTTTTC
RC2-454-TGA-R	CTGCGTGGTTCATCCACTTGGAGTGTTTGTTCTGCTCAAGTAATACAGG
AAP-TGA-TAA-F	GGATACCTCACCCTAAACAACGGGAGTCAGGCAGTAGG
AAP-TGA-TAA-R	CTGACTCCCGTTGTTTAGGGTGAGGTATCCATACTGTGGC
Rep-TGA-TAA-F	ACACTCTCTCTAAAGGAATAAGACAGTGGTGGAAGCTCAAACCTG
Rep-TGA-TAA-R	ACCACTGTCTTATTCCTTTAGAGAGAGTGTCCTCGAGCCAATCTG
RC2-SbfI-R	TTCGATCATTCCTGCAGGTGTAGTTAATGATTAACCCGCC
SbfI-CMV-F	AGGTACCTTCAACCTGCAGGTTGACATTGATTATTGACTAG
Cap-Bsu36-R	TCAGTATTGAGCCTCAGGTGTAGTTAATGATTAACCCGCCATGCTACTTATC
NheI-HTS25-F	GTTTGAGACGGGCGACAGATC
SbfI-HTS25-R	ATATTAATTCCTGCAGGCTCGATCCGCTCGCACCC
EcYRS-NheI-F	TTTGAGGAATCCGCTAGCGCAAGCAGTAACTTGATTAAACAATTGCAAGAG
EcYRS-XhoI-R	AATTCTCGAGTTATTTCCAGCAAATCAGACACTAATTC
NotI-ColE1-R	AATTTAACCTGAGGCGGCCGCAGCAAAAGGCCAGGAAC
KpnI-F1ori-F	TTAATTAAGCTTGGTACCACGCGCCCTGTAGCGGCG
PolyA-KpnI-R	AAATTGGTACCATAGAGCCCACCGC
NotI-SbfI-CMV-F	TTAATGCGGCCGCCTGCAGGGACATTGATTATTGACTAG
Mb-R284-F	CTGGCTAGCGCCACCATGGATAAAAAAC
270G-R	CAGATAGTTGTAGCCAGTCGGGGCAAGCATTGGCC
270G-F	CTTGCCCCGACTGGCTACAACTATCTGCGAAAACTCGATAGGATTTTACC
311G313A-R	CCCATCTGTGCGAAGCCCACCATAGTAAATTCTTCCAGGTGCTCTTTGC
311G313A-F	GAATTTACTATGGTGGGCTTCGCACAGATGGGTTCGGGATGTACTCGGG
Mb-R284-R	AAAGTCGACTTAACGCGTTGAATTCTTACAGATTGGTTGAAATC

Production of ncAA-containing AAV.

The protocol for AAV2 production and purification was described previously[5]. AAV2 was produced by triple transfection (1:1:1 molar ratio) on HEK293T cells with plasmids containing AdHelper genes, suitable AAV genes, and a pair of orthogonal aaRS/tRNA. Specifically, pIDTsmart-RC2-

454-TAG/TGA was used for the incorporation of ncAAs throughout the capsid, pIDTsmart-RC2-ΔVP1-CMV-VP1-454-TAG/TGA was used for the incorporation of ncAAs at single capsid VP1. For the incorporation of two ncAAs to the AAV capsid, 4 plasmids (1:1:1:1 molar ratio) were used: pHelper, pIDTsmart-RC2-delVP1-456-TAG-CMV-VP1-454-TGA, pAAV-WRS-4xWtR-ITR-GFP, pIDTsmart-PLRS-4xLtR were used for transient transfection of HEK293T cells.

For small-scale production of ncAA-containing AAVs, 12 well plates were transfected at 70% confluency. 1.5 µg total DNA/well was mixed with polyethyleneimine (Sigma) in serum-free media (DMEM, HyClone) at RT. After 15 min of incubation, the mixture was added to HEK293T cells. For the incorporation of ncAA into the viral capsid, the growth media was supplemented with 0.5 mM ncAA for the experiment described in Figure 1, 0.2 mM ncAA for the experiment described in Figure 2, 0.4 mM ncAA for the experiment described in Figure 3. Viruses were harvested 3 days post-transfection using AAVPro Extraction Solution Kit (Takara).

For larger-scale production of ncAA-containing AAVs, 15 cm dishes were transfected at 70% confluency. 57 µg total DNAs were mixed with polyethyleneimine (Sigma) in serum-free media (DMEM, HyClone) at RT. After 15 min incubation, the mixture was added to HEK293T cells in media supplemented with 0.5 mM ncAA. Virus was harvested 5 days post-transfection. Media was collected and precipitated with 30% volume of 40% PEG 8000. Cells were lysed by 2 freeze-thaw cycles in a dry ice/ethanol bath. Cell lysate and media were pooled together, and purified using AVB column, following the manufacturer's protocols. The virus was titered using the AAVpro® Titration Kit (for Real-Time PCR) Ver.2 (Takara), following manufacturer's instructions.

Dot blot analysis

AAV2 WT and ncAA-mutants were produced by transient transfection in a 12-well plate of HEK293T cells. Viruses were harvested from cell lysate 3 days post-transfection by Takara kit as mentioned above. The clarified cell-free extract was adjusted to a final concentration of 1 mg/mL and 2 µL of lysates containing each virus and non-treated cell control were spotted in a nitrocellulose membrane (GE Healthcare Life Sciences). The membrane was blocked with 5% non-fat milk with blocking solution (5% nonfat milk and 0.1% Tween 20 (Fisher Scientific) in Tris-buffered saline (TBS)) for 2 h on a shaker at RT. Then, the membrane was incubated with anti-AAV antibodies (A20 clone, ARP American Research) in fresh blocking buffer overnight on a shaker at RT to detect the intact capsids. Next day, the blot was washed 6 times with wash solution (0.1% Tween 20 in TBS). Then, the membrane was incubated with chicken anti-mouse IgG secondary antibody-HRP conjugate (Fisher Scientific) in blocking buffer for 2 h on a shaker at RT, followed by 6 washes with wash buffer. The membrane was developed using SuperSignal West Dura Kit (Fisher Scientific) and the signal was detected by the ChemiDoc MP imaging system (BioRad).

Assaying the infectivity of WT and mutant AAV2

Infectivity was assayed as previously described[1]. In brief, viruses were used to infect HEK293T cells at 90-100% confluency. The transgene expression of EGFP/ luciferase reporter was enhanced by adding 5 mM sodium butyrate (Sigma-Aldrich) in the growth media. After 48 h, cells were imaged using a Zeiss Axio Observer fluorescence microscope (for EGFP) with an XCite Series 120Q light source and Zeiss filter 44 (excitation 475/40 nm, beamsplitter 500 nm, emission

530/50 nm). Media was removed and cells were then lysed with CelLytic M (Sigma). The total EGFP signal was read by a Synergy Neo2 Hybrid Multi-Mode Microplate Reader (BioTek) (488 nm excitation, 530 nm cutoff filter, 532 nm emission). For luciferase, the expression level was assayed by Luciferase Assay System (Promega), following the manufacturer's instructions.

Capsid labeling, site and stoichiometry specific pegylation of AAV2, and SDS-Page

Approximately 10^{10} genome copies of purified AAV2 WT and mutants were used for capsid labeling. For labeling AAV capsid by SPAAC, purified AAV2 WT, VP1-454-LCA, and VP1-454-pAAF viruses were mixed with 20 μM DBCO-TAMRA at room temperature for 30 min. For labeling AAV capsid by IEDDA, purified AAV2 WT and VP1-454-CpK were mixed with 20 μM Tetrazine-Fluorescein at room temperature for 30 min. For labeling AAV capsid by electrochemical reaction (eCLIC), purified AAV2 WT and VP1-454-HTP were labeled with 1.5 mM dialkylaniline-PEG-alkyne for 50 minutes under optimized eCLIC conditions. Excess probes were washed away and Alkyne-functionalized AAVs were fluorophore labelled by copper-catalyzed alkyne-azide cycloaddition for 1 hour with 2 μM rhodamine azide, 500 μM $CuSO_4$, 500 μM TCEP, and 170 μM TBTA ligand before precipitation in 10x volume of ice-cold HPLC-grade acetone. After re-dissolving fluorophore-labelled conjugate in 1x Laemmli buffer, the reaction was analyzed by SDS-PAGE.

Purified AAV2 WT and various mutants containing azide at VP1 or VP1+VP2 or all VP1+VP2+VP3 were nutated with 125 μM DBCO-mPEG-20 kDa (Broadpharm) at RT overnight. On the next day, excess DCBO- mPEG-20 kDa was washed away by acetone precipitation and the conjugation of selected capsid proteins with mPEG-20 kDa was analyzed by SDS-PAGE.

The viruses were heated in SDS-loading buffer for 1 min under reducing condition, then analyzed by 10%-12% SDS-PAGE gel. TAMRA fluorescence was imaged with the appropriate settings on the ChemiDoc MP imaging system (BioRad) (rhodamine setting for Tamra and rhodamine, Alexa 488 setting for Fluorescein). Proteins were stained with SYPRO™ Orange Protein Stain (Thermo Fisher Scientific), followed the manufacturer's instructions, and imaged with Dylight 540.

Protein expression, AAV-protein conjugation and retargeting

AntiHer2-Nanobody (Clone 5F7)-69-AzK expression and purification was done using the previously described methods[9]. The purified nanobody (200 μM) and 50 mM DBCO-PEG12-TCO (Broadpharm) were diluted in 1% DMSO in DPBS (pH = 7.4) to final concentration of 20 μM and 200 μM, respectively. Conjugations were done in 100 μL scale in 0.6 mL tubes by nutating the mixtures at 22°C overnight. The modified nanobody containing TCO functional group was buffer exchanged in DPBS using ultracentrifugal device 0.5 mL 10kDa to remove excess DBCO probes. Concentration was retaken by Pierce™ Coomassie (Bradford) Protein Assay (Thermo Scientific™).

For direct AAV-protein conjugation, the TCO-Nb was diluted in the virus solution containing purified KO.VP1-588-BuTz to the final concentration of 1 μM. Conjugation was done in 0.6 mL tubes by nutating the mixtures at 22 °C for 4 h.

For 2-step AAV-protein conjugation, 50 mM DBCO-Tz (Broadpharm) was diluted in purified AAV KO.VP1-588-AzK to a final concentration of 100 µM. Conjugations were done in 200 µL scale in 0.6 mL tubes by nutating the mixtures at 22°C overnight. The Tz-labeled AAV was buffer exchanged in AAV buffer (1xDPBS, 300 µM NaCl, 0.001% Pluronic F68), using an ultracentrifugal device 4 mL 100kDa to remove excess DBCO probes. TCO-Nb was diluted in the virus solution of Tz-labeled AAV to a final concentration of 1 µM. Conjugation was done in 0.6 mL tubes by nutating the mixtures at 22 °C for 4 h.

SK-BR-3 cells were counted by Bio-Rad TC20™ Automated Cell Counter and 2.5x 10^6 cells were seeded in a 24 well plate (Corning). After 30 hours, cells were transduced with a fixed MOI (125) of AAV2-WT, KO.VP1-588-BuTz, and KO.VP1-588-Nb, along with 5 mM sodium butyrate (Sigma-Aldrich) to enhance the expression of AAV2-encoded transgenes. 48 h post-infection, media was removed, cells were lysed with CelLytic M (Sigma), and luminescence was assayed by Luciferase Assay System (Promega), following the manufacturer's instructions.

Animal study

This Protocol was approved by and was conducted in compliance with Charles River Laboratories (MA) Institutional Animal Care and Use Committee (IACUC).

AAV2 WT, LCA-containing at VP1 without and with PEGylation viruses were sterile filtered by passing through a 0.2 µm, 4 mm filter. The content of endotoxin in the virus samples was assayed and found to be < 1.000 EU/ml using the ToxinSensorTM Chromogenic LAL Endotoxin Assay Kit (Genscript). Animals were prepared and *in vivo* imaging was performed by Charles River Laboratories. All study animals (3 groups of 4 female mice were randomly pooled) received a single dose of 5.00 x 10^{10} gc of AAV2 WT or VP1-LCA or VP1-PEG20K via an intravenous bolus injection into the tail vein. Prior to dose administration, study animals were placed under lamps (within their cages) for a total of 3 –6 minutes for warming. Approximately 15 min before IVIS imaging, each animal received an intraperitoneal injection fixed dose (0.2 mL/mouse) of Luciferin-D on Study Day 29. Blood samples for serum analysis were collected from Groups 1-3 on Day 1 (pre-dose), 14, 28. All animals were euthanized by CO_2 asphyxiation.

ELISA assay to titer the anti-AAV antibodies generated in the animals

About 2 x 10^9 genome copies of AAV2-WT were coated in the Nunc™ Edge™ 96-Well, Nunclon Delta-Treated, Flat-Bottom Microplate (Thermo Fisher Scientific) in PBS at 4 °C on an orbital shaker overnight. On the next day, the coating solution was removed and the plate was washed 3 times with wash solution (0.05% Tween 20 in TBS). The plate was blocked with blocking solution (1% BSA in PBS), incubated at 37 °C on a nutator for 1 h, followed by 4 times washing with wash solution. Standard and mouse serums were diluted in dilution solution (0.1% BSA, 0.05% Tween 20 in PBS) at 20,000 folds for samples. 100 µL was added per well and incubated at 37 °C on a nutator for 1 hour, followed by 3 washes with wash solution. Next, chicken anti-mouse IgG-HRP (Fisher Scientific) (1:2000 dilution in dilution solution) was added per well and the plate was nutated for another hour at 37 °C, followed by 3 washes with wash solution. Then, 100 µL of the 1-Step™ TMB ELISA Substrate Solutions (Thermo Scientific) was added to each microplate well and the plate was incubated at room temperature for 20 min. The color development was

quenched by adding 100 µL of 2 M sulfuric acid to each well, and the absorbance at 450 nm was read by a Synergy Neo2 Hybrid Multi-Mode Microplate Reader (BioTek).

Plasmid maps and sequences

pIDTsmart-PLRS-4xLtR-ITR-GFP

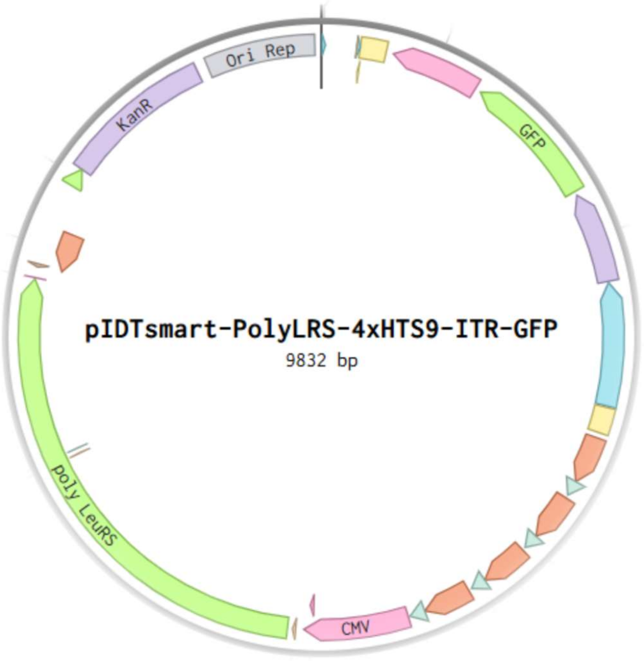

cccgtgtaaaacgacggccagtttatctagtcagcttgattctagctgatcgtggaccggaaggtgagccagtgagttgattgcagtccagttacgctggagtctgaggctcgtcctgaatgat
atgcgaccgccggagggttgcgtttgagacgggcgacagatccagtcgcgctgctctcgtcgatccgctagggcggccgcaaatacctgcaggcagctgcgcgctcgctcgctcactga
ggccgcccgggcaaagcccgggcgtcgggcgacctttggtcgcccggcctcagtgagcgagcgagcgcgcagagagggagtggccaactccatcactaggggttcctgcggccgct
cggtccgcacgtggttacctacaaaatcagaaggacagggaagggagcagtggttcacgcctgtaatcccagcaatttgggaggccaaggtgggtagatcacctgagattaggagttgg
agaccagcctggccaatatggtgaaaccccgtctctaccaaaaaacaaaaattagctgagcctggtcatgcatgcctggaatcccaacaactcgggaggctgaggcaggagaatcg
cttgaacccaggaggcggagattgcagtgagccaagattgtgccactgcactccagcttggttcccaatagaccccgcaggccctacaggttgtcttcccaacttgccccttgctccatacc
acccccctccaccccataatattatagaaggacacctagtcagacaaaatgatgcaacttaattttattaggacaaggctggtgggcactggagtggcaacttccagggccaggagaggc
actggggaggggtcacagggatgccacccgtagatctctcgagcagcgctgctcgaggcaagcttacttgagctcgagatctgagtacttgtacagctcgtccatgccgagagtgatcccg
gcggcggtcacgaactccagcaggaccatgtgatcgcgcttctcgttggggtctttgctcagggcggactgggtgctcaggtagtggttgtcgggcagcagcacggggccgtcgccgatg
ggggtgttctgctggtagtggtcggcgagctgcacgctgccgtcctcgatgttgtggcggatcttgaagttcaccttgatgccgttcttctgcttgtcggccatgatatagacgttgtggctgttgtag
ttgtactccagcttgtgccccaggatgttgccgtcctccttgaagtcgatgcccttcagctcgatgcggttcaccagggtgtcgccctcgaacttcacctcggcgcgggtcttgtagttgccgtcgt
ccttgaagaagatggtgcgctcctggacgtagccttcgggcatggcggacttgaagaagtcgtgctgcttcatgtggtcggggtagcggctgaagcactgcacgccgtaggtcagggtggt
cacgagggtgggccagggcacgggcagcttgccggtggtgcagatgaacttcagggtcagcttgccgtaggtggcatcgccctcgccctcgccggacacgctgaacttgtggccgtttac
gtcgccgtccagctcgaccaggatgggcaccaccccggtgaacagctcctcgcccttgctcaccattcagaattcaatcgatgttcgaatcccaattctttgccaaagtgatgggccagcac
acagaccagcacgttgcccaggagctgtgggaggaagataagaggtatgaacatgattagcaaaagggcctagcttggactcagaataatccagccttatcccaaccataaaataaaa
gcagaatggtagctggattgtagctgctattagcaatatgaaacctcttacatcagttacaatttatatgcagaaatatttatatgcagaaatattgctattgccttaacccagaaattatcactgtt
attctttagaatggtgcaaagaggcatgatacattgtatcattattgccctgaaagaaagagattagggaaagtattagaaataagataaacaaaaaagtatattaaaagaagaaagcattt
tttgtgggcctatagactctataggcggtacttacgtcactcttggcacggggaatccgcgttccaatgcaccgttcccggccgggattcgaatccgcggaggctggatcggtcccggtgtctt
ctatggaggtcaaaacagcgtggatggcgtctccaggcgatctgacggttcactaaacgagctctgcttatatagacctcccaccgtacacgcctaccgcccatttgcgtcaatggggcgg
agttgttacgacattttggaaagtcccgttgattttggtgcaaaacaaactcccattgacgtcaatggggtggagacttggaaatccccgtgagtcaaaccgctatccacgcccattgatgtact
gccaaaaccgcatcaccatggtaatagcgatgactaatacgtagatgtactgccaagtaggaaagtcccataaggtcatgtactgggcataatgccaggcgggccatttaccgtcattgac
gtcaataggggggcgtacttggcatatgatacacttgatgtactgccaagtgggcagtttaccgtaaatactccacccattgacgtcaatggaaagtccctattgacgttactatgggaacatac
gtcattattgacgtcaatgggcgggggtcgttgggcggtcagccaggcgggccatttaccgtaagttatgtaacgcggaactccatatatgggctatgaactaatgaccccgtaattgattact
attaataactagctccacgcgtgcggccgcaggaacccctagtgatggagttggccactccctctctgcgcgctcgctcgctcactgaggccgggcgaccaaaggtcgcccgacgcccg
ggctttgcccgggcggcctcagtgagcgagcgagcgcgcagctgcctgcaggctcgatccgctcgcacccctaggggtacctcgggcaggaagagggcctatttcccatgattccttcat
atttgcatatacgatacaaggctgttagagagataattagaattaatttgactgtaaacacaaagatattagtacaaaatacgtgacgtagaaagtaataatttcttgggtagtttgcagttttaa
aattatgttttaaaatggactatcatatgcttaccgtaacttgaaagtatttcgatttcttggctttatatatcttgtggaaaggacgaaacaccgcccgtatggtggaatcggtagacacaaggga
ttctaaatccctcggcgttcgcgctgtgcgggttcaagtcccgctgcgggcatttttgctaggggtacctcgggcaggaagagggcctatttcccatgattccttcatatttgcatatacgataca

aggctgttagagagataattagaattaatttgactgtaaacacaaagatattagtacaaaatacgtgacgtagaaagtaataatttcttgggtagtttgcagttttaaaattatgttttaaaatgga
ctatcatatgcttaccgtaacttgaaagtatttcgatttcttggctttatatatcttgtggaaaggacgaaacaccgcccgtatggtggaatcggtagacacaagggattctaaatccctcggcgtt
cgcgctgtgcgggttcaagtcccgctgcgggcatttttgctaggggtacctcgggcaggaagagggcctatttcccatgattccttcatatttgcatatacgatacaaggctgttagagagata
attagaattaatttgactgtaaacacaaagatattagtacaaaatacgtgacgtagaaagtaataatttcttgggtagtttgcagttttaaaattatgttttaaaatggactatcatatgcttaccgta
acttgaaagtatttcgatttcttggctttatatatcttgtggaaaggacgaaacaccgcccgtatggtggaatcggtagacacaagggattctaaatccctcggcgttcgcgctgtgcgggttca
agtcccgctgcgggcatttttgctaggggtacctcgggcaggaagagggcctatttcccatgattccttcatatttgcatatacgatacaaggctgttagagagataattagaattaatttgactg
taaacacaaagatattagtacaaaatacgtgacgtagaaagtaataatttcttgggtagtttgcagttttaaaattatgttttaaaatggactatcatatgcttaccgtaacttgaaagtatttcgattt
cttggctttatatatcttgtggaaaggacgaaacaccgcccgtatggtggaatcggtagacacaagggattctaaatccctcggcgttcgcgctgtgcgggttcaagtcccgctgcgggcattt
ttgctagttattaatagtaatcaattacggggtcattagttcatagcccatatatggagttccgcgttacataacttacggtaaatggcccgcctggctgaccgcccaacgacccccgcccattg
acgtcaataatgacgtatgttcccatagtaacgccaatagggactttccattgacgtcaatgggtggactatttacggtaaactgcccacttggcagtacatcaagtgtatcatatgccaagtac
gccccctattgacgtcaatgacggtaaatggcccgcctggcattatgcccagtacatgaccttatgggactttcctacttggcagtacatctacgtattagtcatcgctattaccatggtgatgcg
gttttggcagtacatcaatgggcgtggatagcggtttgactcacggggatttccaagtctccaccccattgacgtcaatgggagtttgttttggcaccaaaatcaacgggactttccaaaatgtc
gtaacaactccgccccattgacgcaaatgggcggtaggcgtgtacggtgggaggtctatataagcagagctctctggctaactagagaacccactgcttactggcttatcgaaattaatacg
actcactatagggagacccaagctggctagcgccaccatggaagagcaataccgcccggaagagatagaatccaaagtacagcttcattgggatgagaagcgcacatttgaagtaac
cgaagacgagagcaaagagaagtattactgcctgtctatcctcccctatccttctggtcgactacacatggccacgtacgtaactacaccatcggtgacgtgatcgcccgctaccagcgta
tgctgggcaaaaacgtcctgcagccgatcggctgggacgcgtttggtctgcctgcggaaggcgcggcggtgaaaaacaacaccgctccggcaccgtggacgtacgacaacatcgcgt
atatgaaaaaccagctcaaaatgctgggctttggttatgactggagccgcgagctggcaacctgtacgccggaatactaccgttgggaacagaaattcttcaccgagctgtataaaaaag
gcctggtatataagaagacttctgcggtcaactggtgtccgaacgaccagaccgtactggcgaacgaacaagttatcgacggctgctgctggcgctgcgataccaaagttgaacgtaaag
agatcccgcagtggtttatcaaaatcactgcttacgctgacgagctgctcaacgatctggataaactggatcactggccagacaccgttaaaaccatgcagcgtaactggatcggtcgttcc
gaaggcgtggagatcaccttcaacgttaacgactatgacaacacgctgaccgtttacactacccgcccggacgcgtttatgggttgtacctacctggcggtagctgcgggtcatccgctggc
gcagaaagcggcggaaaataatcctgaactggcggcctttattgacgaatgccgtaacaccaaagttgccgaagctgaaatggcgacgatggagaaaaaaggcgtcgatactggcttt
aaagcggttcacccattaacgggcgaagaaattcccgtttgggcagcaaacttcgtattgatggagtacggcacgggcgcagttatggcggtaccggggcacgaccagcgcgactacg
agtttgcctctaaatacggcctgaacatcaaaccggttatcctggcagctgacggctctgagccagatctttctcagcaagccctgactgaaaaaggcgtgctgttcaactctggcgagttca
acggtcttgaccatgaagcggccttcaacgccatcgccgataaactgactgcgatgggcgttggcgagcgtaaagtgaactaccgcctgcgcgactggggtgtttcccgtcagcgttactg
ggcgcgccgattccgatggtgactctagaagacggtaccgtaatgccgaccccggacgaccagctgccggtgatcctgccggaagatgtggtaatggacggcattaccagcccgatta
aagcagatccggagtgggcgaaaactaccgttaacggtatgccagcactgcgtgaaaccgacactttcgacacctttatggagtcctcctggatctatgcgcgctacacttgcccgcagta
caaagaaggtatgctggattccgaagcggctaactactggctgccggtggatatcgctattggtggtattgaacacgccattatgggtctgctctacttccgcttcttccacaaactgatgcgtg
atgcaggcatggtgaactctgacgaaccagcgaaacagttgctgtgtcagggtatggtgctggcagatgccttctactatgttggcgaaaacggcgaacgtaactgggtttccccggttgat
gctatcgttgaacgtgacgagaaaggccgtatcgtgaaagcgaaagatgcggcaggccatgaactggtttataccggcatgagcaaaatgtccaagtcgaagaacaacggtatcgacc
cgcaggtgatggttgaacgttacggcgcggacaccgttcgtctgtttatgatgtttgcttctccggctgatatgactctcgaatggcaggaatccggtgtggaaggggctaaccgcttcctgaaa
cgtgtctggaaactggtttacgagcacacagcaaaaggtgatgttgcggcactgaacgttgatgcgctgactgaaaatcagaaagcgctgcgtcgcgatgtgcataaaacgatcgctaaa
gtgaccgatgatatcggccgtcgtcagaccttcaacaccgcaattgcggcgattatggagctgatgaacaaactggcgaaagcaccaaccgatggcgagcaggatcgcgctctgatgc
aggaagcactgctggccgttgtccgtatgcttaacccgttcaccccgcacatctgcttcacgctgtggcaggaactgaaaggcgaaggcgatatcgacaacgcgccgtggccggttgctg
acgaaaaagcgatggtggaagactccacgctggtcgtggtgcaggttaacggtaaagtccgtgccaaaatcaccgttccggtggacgcaacggaagaacaggttcgcgaacgtgctg
gccaggaacatctggtagcaaaatatcttgatggcgttactgtacgtaaagtgatttacgtaccaggtaaactcctcaatctggtcgttggcgggcccgtttaagaattcaacgcgttaagtcg
actttaactcgagtctagagggcccgtttaaacccgctgatcagcctcgactgtgccttctagttgccagccatctgttgtttgcccctcccccgtgccttccttgaccctggaaggtgccactccc
actgtcctttcctaataaaatgaggaaattgcatcgcattgtctgagtaggtgtcattctattctggggggtggggtggggcaggacagcaagggggaggattgggaagacaatagcaggc
atgctggggatgcggtgggctctatggcttctgaggcggaaagaaccctaggggtgcgagcggatcgagcagtgtcgatcactactggaccgcgagctgtgctgcgacccgtgatcttac
ggcattatacgtatgatcggtccacgatcagctagattatctagtcagcttgatgtcatagctgtttcctgaggctcaatactgaccatttaaatcatacctgacctccatagcagaaagtcaaaa
gcctccgaccggaggcttttgacttgatcggcacgtaagaggttccaactttcaccataatgaaataagatcactaccgggcgtatttttgagttatcgagattttcaggagctaaggaagcta
aaatgagccatattcaacgggaaacgtcttgcttgaagccgcgattaaattccaacatggatgctgatttatatgggtataaatgggctcgcgataatgtcgggcaatcaggtgcgacaatct
atcgattgtatgggaagcccgatgcgccagagttgtttctgaaacatggcaaaggtagcgttgccaatgatgttacagatgagatggtcaggctaaactggctgacggaatttatgcctcttcc
gaccatcaagcattttatccgtactcctgatgatgcatggttactcaccactgcgatccagggaaaacagcattccaggtattagaagaatatcctgattcaggtgaaaatattgttgatgcgc
tggcagtgttcctgcgccggttgcattcgattcctgtttgtaattgtccttttaacggcgatcgcgtatttcgtctcgctcaggcgcaatcacgaatgaataacggtttggttggtgcgagtgattttga
tgacgagcgtaatggctggcctgttgaacaagtctggaaagaaatgcataaactcttgccattctcaccggattcagtcgtcactcatggtgatttctcacttgataaccttatttttgacgaggg
gaaattaataggttgtattgatgttggacgagtcggaatcgcagaccgataccaggatcttgccatcctatggaactgcctcggtgagttttctccttcattacagaaacggctttttcaaaaatat
ggtattgataatcctgatatgaataaattgcagtttcacttgatgctcgatgagtttttctaatgaggacctaaatgtaatcacctggctcaccttcgggtgggcctttctgcgttgctggcgtttttcca
taggctccgcccccctgacgagcatcacaaaaatcgatgctcaagtcagaggtggcgaaacccgacaggactataaagataccaggcgtttccccctggaagctccctcgtgcgctctc
ctgttccgaccctgccgcttaccggatacctgtccgcctttctcccttcgggaagcgtggcgctttctcatagctcacgctgtaggtatctcagttcggtgtaggtcgttcgctccaagctgggctgt
gtgcacgaacccccgttcagcccgaccgctgcgccttatccggtaactatcgtcttgagtccaacccggtaagacacgacttatcgccactggcagcagccactggtaacaggattagc
agagcgaggtatgtaggcggtgctacagagttcttgaagtggtggcctaactacggctacactagaagaacagtatttggtatctgcgctctgctgaagccagttacctcggaaaaagagtt
ggtagctcttgatccggcaaacaaaccaccgctggtagcggtggtttttttgtttgcaagcagcagattacgcgcagaaaaaaaggatctcaagaagatcctttgatcttttctacggggtctgacgctcagtggaacgaaaactcacgttaagggattttggtcatgagattatcaaaaaggatcttcacctagatccttttaaattaaaaatgaagttttaaatcaatctaaagtatatatgagtaaacttggtctgacagttaccaatgcttaatcagtgaggcacctatctcagcgatctgtctatttcgttcatccatagttgcctgactccccgtcgtgtagataactacgatacgggagggcttaccatctggccccagtgctgcaatgataccgcgagacccacgctcaccggctccagatttatcagcaataaaccagccagccggaagggccgagcgcagaagtggtcctgcaactttatccgcctccatccagtctattaattgttgccgggaagctagagtaagtagttcgccagttaatagtttgcgcaacgttgttgccattgctacaggcatcgtggtgtcacgctcgtcgtttggtatggcttcattcagctccggttcccaacgatcaaggcgagttacatgatcccccatgttgtgcaaaaaagcggttagctccttcggtcctccgatcgttgtcagaagtaagttggccgcagtgttatcactcatggttatggcagcactgcataattctcttactgtcatgccatccgtaagatgcttttctgtgactggtgagtactcaaccaagtcattctgagaatagtgtatgcggcgaccgagttgctcttgcccggcgtcaatacgggataataccgcgccacatagcagaactttaaaagtgctcatcattggaaaacgttcttcggggcgaaaactctcaaggatcttaccgctgttgagatccagttcgatgtaacccactcgtgcacccaactgatcttcagcatcttttactttcaccagcgtttctgggtgagcaaaaacaggaaggcaaaatgccgcaaaaaagggaataagggcgacacggaaatgttgaatactcatactcttcctttttcaatattattgaagcatttatcagggttattgtctcatgagcggatacatatttgaatgtatttagaaaaataaacaaataggggttccgcgcacatttccccgaaaagtgccacctgacgtc

pIDTsmart-YRS-4xYtR-ITR-GFP

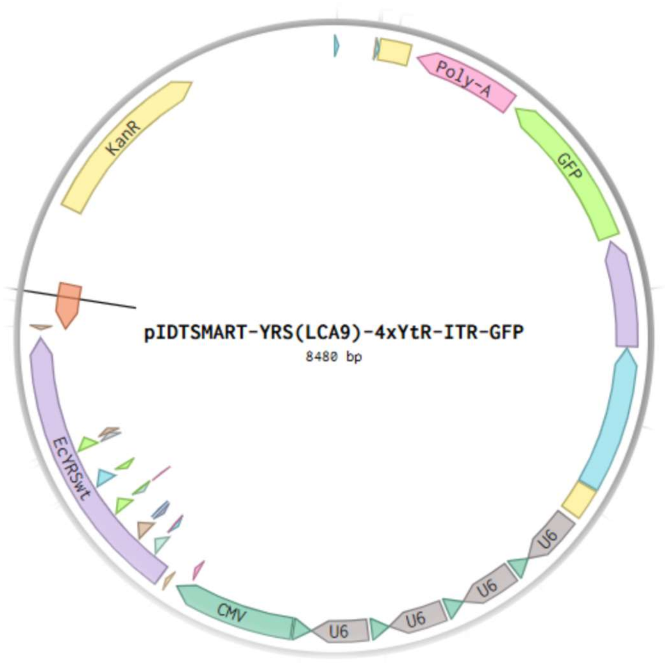

cccgtgtaaaacgacggccagtttatctagtcagcttgattctagctgatcgtggaccggaaggtgagccagtgagttgattgcagtccagttacgctggagtctgaggctcgtcctgaatgat
atgcgaccgccggagggttgcgtttgagacgggcgacagatccagtcgcgctgctctcgtcgatccgctagggcggccgcaaatacctgcaggcagctgcgcgctcgctcgctcactga
ggccgcccgggcaaagcccgggcgtcgggcgacctttggtcgcccggcctcagtgagcgagcgagcgcgcagagagggagtggccaactccatcactaggggttcctgcggccgct
cggtccgcacgtggttacctacaaaatcagaaggacagggaagggagcagtggttcacgcctgtaatcccagcaatttgggaggccaaggtgggtagatcacctgagattaggagttgg
agaccagcctggccaatatggtgaaaccccgtctctaccaaaaaacaaaaattagctgagcctggtcatgcatgcctggaatcccaacaactcgggaggctgaggcaggagaatcg
cttgaacccaggaggcggagattgcagtgagccaagattgtgccactgcactccagcttggttcccaatagaccccgcaggccctacaggttgtcttcccaacttgccccttgctccatacc
accccccctccaccccataatattatagaaggacacctagtcagacaaaatgatgcaacttaattttattaggacaaggctggtgggcactggagtggcaacttccagggccaggagaggc
actggggaggggtcacagggatgccacccgtagatctctcgagcagcgctgctcgaggcaagcttacttgagctcgagatctgagtacttgtacagctcgtccatgccgagagtgatcccg
gcggcggtcacgaactccagcaggaccatgtgatcgcgcttctcgttggggtctttgctcagggcggactgggtgctcaggtagtggttgtcgggcagcagcacggggccgtcgccgatg
ggggtgttctgctggtagtggtcggcgagctgcacgctgccgtcctcgatgttgtggcggatcttgaagttcaccttgatgccgttcttctgcttgtcggccatgatatagacgttgtggctgttgtag
ttgtactccagcttgtgccccaggatgttgccgtcctccttgaagtcgatgcccttcagctcgatgcggttcaccagggtgtcgccctcgaacttcacctcggcgcgggtcttgtagttgccgtcgt
ccttgaagaagatggtgcgctcctggacgtagccttcgggcatggcggacttgaagaagtcgtgctgcttcatgtggtcggggtagcggctgaagcactgcacgccgtaggtcagggtggt
cacgagggtgggccagggcacgggcagcttgccggtggtgcagatgaacttcagggtcagcttgccgtaggtggcatcgccctcgccctcgccggacacgctgaacttgtggccgtttac
gtcgccgtccagctcgaccaggatgggcaccaccccggtgaacagctcctcgcccttgctcaccattcagaattcaatcgatgttcgaatcccaattctttgccaaagtgatgggccagcac
acagaccagcacgttgcccaggagctgtgggaggaagataagaggtatgaacatgattagcaaaagggcctagcttggactcagaataatccagccttatcccaaccataaaataaaa
gcagaatggtagctggattgtagctgctattagcaatatgaaacctcttacatcagttacaatttatatgcagaaatatttatatgcagaaatattgctattgccttaacccagaaattatcactgtt
attctttagaatggtgcaaagaggcatgatacattgtatcattattgccctgaaagaaagagattagggaaagtattagaaataagataaacaaaaaagtatattaaaagaagaaagcattt
tttgtgggcctatagactctataggcggtacttacgtcactcttggcacggggaatccgcgttccaatgcaccgttcccggccgggattcgaatccgcggaggctggatcggtcccggtgtctt
ctatggaggtcaaaacagcgtggatggcgtctccaggcgatctgacggttcactaaacgagctctgcttatatagacctcccaccgtacacgcctaccgcccatttgcgtcaatggggcgg
agttgttacgacattttggaaagtcccgttgattttggtgcaaaacaaactcccattgacgtcaatggggtggagacttggaaatccccgtgagtcaaaccgctatccacgcccattgatgtact
gccaaaaccgcatcaccatggtaatagcgatgactaatacgtagatgtactgccaagtaggaaagtcccataaggtcatgtactgggcataatgccaggcgggccatttaccgtcattgac
gtcaatagggggcgtacttggcatatgatacacttgatgtactgccaagtgggcagtttaccgtaaatactccacccattgacgtcaatggaaagtccctattgacgttactatgggaacatac
gtcattattgacgtcaatgggcgggggtcgttgggcggtcagccaggcgggccatttaccgtaagttatgtaacgcggaactccatatatgggctatgaactaatgaccccgtaattgattact
attaataactagctccacgcgtgcggccgcaggaacccctagtgatggagttggccactccctctctgcgcgctcgctcgctcactgaggccgggcgaccaaaggtcgcccgacgcccg
ggctttgcccgggcggcctcagtgagcgagcgagcgcgcagctgcctgcaggctcgatccgctcgcacccctaggggtacctcgggcaggaagagggcctatttcccatgattccttcat
atttgcatatacgatacaaggctgttagagagataattagaattaatttgactgtaaacacaaagatattagtacaaaatacgtgacgtagaaagtaataatttcttgggtagtttgcagttttaa
aattatgttttaaaatggactatcatatgcttaccgtaacttgaaagtatttcgatttcttggctttatatatcttgtggaaaggacgaaacaccggtggggttcccgagcggccaaagggagcag
actctaaatctgccgtcacagacttcgaaggttcgaatccttcccccaccatttttgctaggggtacctcgggcaggaagagggcctatttcccatgattccttcatatttgcatatacgatacaa
ggctgttagagagataattagaattaatttgactgtaaacacaaagatattagtacaaaatacgtgacgtagaaagtaataatttcttgggtagtttgcagttttaaaattatgttttaaaatggact
atcatatgcttaccgtaacttgaaagtatttcgatttcttggctttatatatcttgtggaaaggacgaaacaccggtggggttcccgagcggccaaagggagcagactctaaatctgccgtcac

agacttcgaaggttcgaatccttcccccaccatttttgctaggggtacctcgggcaggaagagggcctatttcccatgattccttcatatttgcatatacgatacaaggctgttagagagataatt
agaattaatttgactgtaaacacaaagatattagtacaaaatacgtgacgtagaaagtaataatttcttgggtagtttgcagttttaaaattatgttttaaaatggactatcatatgcttaccgtaact
tgaaagtatttcgatttcttggctttatatatcttgtggaaaggacgaaacaccggtggggttcccgagcggccaaagggagcagactctaaatctgccgtcacagacttcgaaggttcgaat
ccttcccccaccatttttgctaggggtacctcgggcaggaagagggcctatttcccatgattccttcatatttgcatatacgatacaaggctgttagagagataattagaattaatttgactgtaa
acacaaagatattagtacaaaatacgtgacgtagaaagtaataatttcttgggtagtttgcagttttaaaattatgttttaaaatggactatcatatgcttaccgtaacttgaaagtatttcgatttctt
ggctttatatatcttgtggaaaggacgaaacaccggtggggttcccgagcggccaaagggagcagactctaaatctgccgtcacagacttcgaaggttcgaatccttcccccaccatttttg
ctagttattaatagtaatcaattacggggtcattagttcatagcccatatatggagttccgcgttacataacttacggtaaatggcccgcctggctgaccgcccaacgacccccgcccattgac
gtcaataatgacgtatgttcccatagtaacgccaatagggactttccattgacgtcaatgggtggactatttacggtaaactgcccacttggcagtacatcaagtgtatcatatgccaagtacgc
cccctattgacgtcaatgacggtaaatggcccgcctggcattatgcccagtacatgaccttatgggactttcctacttggcagtacatctacgtattagtcatcgctattaccatggtgatgcggttt
tggcagtacatcaatgggcgtggatagcggtttgactcacggggatttccaagtctccaccccattgacgtcaatgggagtttgttttggcaccaaaatcaacgggactttccaaaatgtcgta
acaactccgccccattgacgcaaatgggcggtaggcgtgtacggtgggaggtctatataagcagagctctctggctaactagagaacccactgcttactggcttatcgaaattaatacgact
cactatagggagacccaagctggctagcgccaccatggcaagcagtaacttgattaaacaattgcaagagcgggggctggtagcccaggtgacggacgaggaagcgttagcagagc
gactggcgcaaggcccgatcgcgctcggctgcggcttcgatcctaccgctgacagcttgcatttggggcatcttgttccattgttatgcctgaaacgcttccagcaggcgggccacaagccg
gttgcggtggtaggcggcgcgacgggtctgattggcgacccgagcttcaaagctgccgagcgtaagctgaacaccgaagaaactgttcaggagtgggtggacaaaatccgtaagcag
gttgccccgttcctcgatttcgactgtggagaaaactctgctatcgcggcgaacaactatgactggttcggcaatatgaatgtgctgaccttcctgcgcgatattggcaaacacttctccgttaac
cagatgatcaacaaagaagcggttaagcagcgtctcaaccgtgaagatcaggggatttcgttcactgagttttcctacaacctgttgcagggttattgttacgcctgttgtaacaaacagtacg
gtgtggtgctgcaaattggtggttctgaccagtggggtaacatcacttctggtatcgacctgacccgtcgtctgcatcagaatcaggtgtttggcctgaccgttccgctgatcactaaagcagat
ggcaccaaatttggtaaaactgaaggcggcgcagtctggttagatccgaagaaaaccagcccgtacaaattctaccagttctggatcaacactgcgcgtgccgacgtttaccgcttcctga
agttcttcacctttatgagcattgaagagatcaacgccctggaagaagaagataaaaacagcggtaaagcaccgcgcgcccagtatgtactggcggagcaggtgactcgtctggttcacg
gtgaagaaggtttacaggcggcaaaacgtattaccgaatgcctgttcagcggttctttgagtgcgctgagtgaagcggacttcgaacagctggcgcaggacggcgtaccgatggttgagat
ggaaaagggcgcagacctgatgcaggcactggtcgattctgaactgcaaccttcccgtggtcaggcacgtaaaactatcgcctccaatgccatcaccattaacggtgaaaaacagtccg
atcctgaatacttctttaaagaagaagatcgtctgtttggtcgttttaccttactgcgtcgcggtaaaaagaattactgtctgatttgctggaaataactcgagtctagagggcccgtttaaacccg
ctgatcagcctcgactgtgccttctagttgccagccatctgttgtttgcccctcccccgtgccttccttgaccctggaaggtgccactcccactgtcctttcctaataaaatgaggaaattgcatcgc
attgtctgagtaggtgtcattctattctggggggtggggtggggcaggacagcaagggggaggattgggaagacaatagcaggcatgctggggatgcggtgggctctatggcttctgagg
cggaaagaaccctaggggtgcgagcggatcgagcagtgtcgatcactactggaccgcgagctgtgctgcgacccgtgatcttacggcattatacgtatgatcggtccacgatcagctaga
ttatctagtcagcttgatgtcatagctgtttcctgaggctcaatactgaccatttaaatcatacctgacctccatagcagaaagtcaaaagcctccgaccggaggcttttgacttgatcggcacgt
aagaggttccaactttcaccataatgaaataagatcactaccgggcgtatttttgagttatcgagattttcaggagctaaggaagctaaaatgagccatattcaacgggaaacgtcttgcttg
aagccgcgattaaattccaacatggatgctgatttatatgggtataaatgggctcgcgataatgtcgggcaatcaggtgcgacaatctatcgattgtatgggaagcccgatgcgccagagttg
tttctgaaacatggcaaaggtagcgttgccaatgatgttacagatgagatggtcaggctaaactggctgacggaatttatgcctcttccgaccatcaagcattttatccgtactcctgatgatgc
atggttactcaccactgcgatccagggaaaacagcattccaggtattagaagaatatcctgattcaggtgaaaatattgttgatgcgctggcagtgttcctgcgccggttgcattcgattcctgt
ttgtaattgtccttttaacggcgatcgcgtatttcgtctcgctcaggcgcaatcacgaatgaataacggtttggttggtgcgagtgattttgatgacgagcgtaatggctggcctgttgaacaagtct
ggaaagaaatgcataaactcttgccattctcaccggattcagtcgtcactcatggtgatttctcacttgataaccttatttttgacgaggggaaattaataggttgtattgatgttggacgagtcgg
aatcgcagaccgataccaggatcttgccatcctatggaactgcctcggtgagttttctccttcattacagaaacggctttttcaaaaatatggtattgataatcctgatatgaataaattgcagtttc
acttgatgctcgatgagtttttctaatgaggacctaaatgtaatcacctggctcaccttcgggtgggcctttctgcgttgctggcgtttttccataggctccgcccccctgacgagcatcacaaaaa
tcgatgctcaagtcagaggtggcgaaacccgacaggactataaagataccaggcgtttccccctggaagctccctcgtgcgctctcctgttccgaccctgccgcttaccggatacctgtccg
cctttctcccttcgggaagcgtggcgctttctcatagctcacgctgtaggtatctcagttcggtgtaggtcgttcgctccaagctgggctgtgtgcacgaaccccccgttcagcccgaccgctgc
gccttatccggtaactatcgtcttgagtccaacccggtaagacacgacttatcgccactggcagcagccactggtaacaggattagcagagcgaggtatgtaggcggtgctacagagttctt
gaagtggtggcctaactacggctacactagaagaacagtatttggtatctgcgctctgctgaagccagttacctcggaaaaagagttggtagctcttgatccggcaaacaaaccaccgctg
gtagcggtggtttttttgtttgcaagcagcagattacgcgcagaaaaaaaggatctcaagaagatcctttgatcttttctaccgaagaaaggccca

pAAV-WRS-4xWtR-ITR-GFP

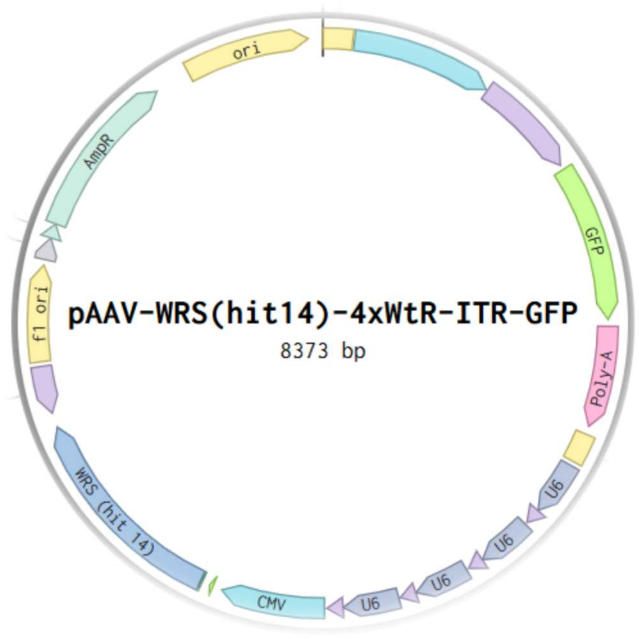

cctgcaggcagctgcgcgctcgctcgctcactgaggccgcccgggcaaagcccgggcgtcgggcgacctttggtcgcccggcctcagtgagcgagcgagcgcgcagagagggagtg
gccaactccatcactaggggttcctgcggccgcacgcgtggagctagttattaatagtaatcaattacggggtcattagttcatagcccatatatggagttccgcgttacataacttacggtaaa
tggcccgcctggctgaccgcccaacgacccccgcccattgacgtcaataatgacgtatgttcccatagtaacgtcaatagggactttccattgacgtcaatgggtggagtatttacggtaaac
tgcccacttggcagtacatcaagtgtatcatatgccaagtacgccccctattgacgtcaatgacggtaaatggcccgcctggcattatgcccagtacatgaccttatgggactttcctacttggc
agtacatctacgtattagtcatcgctattaccatggtgatgcggttttggcagtacatcaatgggcgtggatagcggtttgactcacggggatttccaagtctccaccccattgacgtcaatggga
gtttgttttgcaccaaaatcaacgggactttccaaaatgtcgtaacaactccgccccattgacgcaaatgggcggtaggcgtgtacggtgggaggtctatataagcagagctcgtttagtgaa
ccgtcagatcgcctggagacgccatccacgctgttttgacctccatagaagacaccgggaccgatccagcctccgcggattcgaatcccggccgggaacggtgcattggaacgcggatt
ccccgtgccaagagtgacgtaagtaccgcctatagagtctataggcccacaaaaaatgctttcttcttttaatatacttttttgtttatcttatttctaatactttccctaatctctttctttcagggcaata
atgatacaatgtatcatgcctctttgcaccattctaaagaataacagtgataatttctgggttaaggcaatagcaatatttctgcatataaatatttctgcatataaattgtaactgatgtaagaggttt
catattgctaatagcagctacaatccagctaccattctgcttttattttatggttgggataaggctggattattctgagtccaagctaggcccttttgctaatcatgttcatacctcttatcttcctcccac
agctcctgggcaacgtgctggtctgtgtgctggcccatcactttggcaaagaattgggattcgaacatcgattgaattctgaatggtgagcaagggcgaggagctgttcaccggggtggtgc
ccatcctggtcgagctggacggcgacgtaaacggccacaagttcagcgtgtccggcgagggcgagggcgatgccacctacggcaagctgaccctgaagttcatctgcaccaccggca
agctgcccgtgccctggcccaccctcgtgaccaccctgacctacggcgtgcagtgcttcagccgctaccccgaccacatgaagcagcacgacttcttcaagtccgccatgcccgaaggct
acgtccaggagcgcaccatcttcttcaaggacgacggcaactacaagacccgcgccgaggtgaagttcgagggcgacaccctggtgaaccgcatcgagctgaagggcatcgacttca
aggaggacggcaacatcctggggcacaagctggagtacaactacaacagccacaacgtctatatcatggccgacaagcagaagaacggcatcaaggtgaacttcaagatccgcca
caacatcgaggacggcagcgtgcagctcgccgaccactaccagcagaacacccccatcggcgacggccccgtgctgctgcccgacaaccactacctgagcacccagtccgccctga
gcaaagaccccaacgagaagcgcgatcacatggtcctgctggagttcgtgaccgccgccgggatcactctcggcatggacgagctgtacaagtactcagatctcgagctcaagtaagct
tgcctcgagcagcgctgctcgagagatctacgggtggcatccctgtgacccctccccagtgcctctcctggccctggaagttgccactccagtgcccaccagccttgtcctaataaaattaag
ttgcatcattttgtctgactaggtgtccttctataatattatggggtggaggggggtggtatggagcaaggggcaagttgggaagacaacctgtagggcctgcggggtctattgggaaccaag
ctggagtgcagtggcacaatcttggctcactgcaatctccgcctcctgggttcaagcgattctcctgcctcagcctcccgagttgttgggattccaggcatgcatgaccaggctcagctaattttt
gttttttggtagagacggggtttcaccatattggccaggctggtctccaactcctaatctcaggtgatctacccaccttggcctcccaaattgctgggattacaggcgtgaaccactgctcccttc
cctgtccttctgattttgtaggtaaccacgtgcggaccgagcggccgcaggaacccctagtgatggagttggccactccctctctgcgcgctcgctcgctcactgaggccgggcgaccaaa
ggtcgcccgacgcccgggctttgcccgggcggcctcagtgagcgagcgagcgcgcagctgcctgcaggctcgatccgctcgcaccctaggtcgggcaggaagagggcctatttcccat
gattccttcatatttgcatatacgatacaaggctgttagagagataattagaattaatttgactgtaaacacaaagatattagtacaaaatacgtgacgtagaaagtaataatttcttgggtagttt
gcagttttaaaattatgttttaaaatggactatcatatgcttaccgtaacttgaaagtatttcgatttcttggctttatatatcttgtggaaaggacgaaacaccaggggcgtagttcaattggtagag
caccggtcttcaaaaccgggtgttgggagttcgagtctctccgcccctgccatttttttgctaggtcgggcaggaagagggcctatttcccatgattccttcatatttgcatatacgatacaaggctg
ttagagagataattagaattaatttgactgtaaacacaaagatattagtacaaaatacgtgacgtagaaagtaataatttcttgggtagtttgcagttttaaaattatgttttaaaatggactatcat
atgcttaccgtaacttgaaagtatttcgatttcttggctttatatatcttgtggaaaggacgaaacaccaggggcgtagttcaattggtagagcaccggtcttcaaaaccgggtgttgggagttcg
agtctctccgcccctgccatttttgctaggtcgggcaggaagagggcctatttcccatgattccttcatatttgcatatacgatacaaggctgttagagagataattagaattaatttgactgtaaa
cacaaagatattagtacaaaatacgtgacgtagaaagtaataatttcttgggtagtttgcagttttaaaattatgttttaaaatggactatcatatgcttaccgtaacttgaaagtatttcgatttcttg
gctttatatatcttgtggaaaggacgaaacaccaggggcgtagttcaattggtagagcaccggtcttcaaaaccgggtgttgggagttcgagtctctccgcccctgccatttttgctaggtcgg

gcaggaagagggcctatttcccatgattccttcatatttgcatatacgatacaaggctgttagagagataattagaattaatttgactgtaaacacaaagatattagtacaaaatacgtgacgta
gaaagtaataatttcttgggtagtttgcagttttaaaattatgttttaaaatggactatcatatgcttaccgtaacttgaaagtatttcgatttcttggctttatatatcttgtggaaaggacgaaacacc
aggggcgtagttcaattggtagagcaccggtcttcaaaaccgggtgttgggagttcgagtctctccgcccctgccatttttgctagttattaatagtaatcaattacggggtcattagttcatagc
ccatatatggagttccgcgttacataacttacggtaaatggcccgcctggctgaccgcccaacgacccccgcccattgacgtcaataatgacgtatgttcccatagtaacgccaatagggac
tttccattgacgtcaatgggtggactatttacggtaaactgcccacttggcagtacatcaagtgtatcatatgccaagtacgccccctattgacgtcaatgacggtaaatggcccgcctggcatt
atgcccagtacatgaccttatgggactttcctacttggcagtacatctacgtattagtcatcgctattaccatggtgatgcggttttggcagtacatcaatgggcgtggatagcggtttgactcacg
gggatttccaagtctccaccccattgacgcaaatgggcggtaggcgtgtacggtgggaggtctatataagcagagctctctggctaactagagaacccactgcttactggcttatcgaaatta
atacgactcactatagggagacccaagctggctagcgtttaaacttaagcttgccgccaccatgactaagcccatcgtttttgctggcgcacagccctcaggtgaattgaccattggtaacta
catgggtgcgctgcgtcagtgggtaaacatgcaggatgactaccattgcatttactgtatcgttgaccaacacgcgatcaccgtgcgccaggatgcacagaagctgcgtaaagcgacgct
ggatacgctggccttgtatctggcttgtggtatcgatcctgagaaaagcaccatttttgttcagtcccacgtgccggaacatgcacagttaggctgggcactgaactgctatacctacttcggcg
aactgagtcgcatgacgcagtttaaagataaatctgcgcgttatgccgagaacatcaacgctggtctgtttgactatccggtgctgatggcagcggacatcctgctgtatcaaactaatctggg
tccttgtggtgaagaccagaaacagcacctcgaactgagccgcgatattgcccagcgtttcaacgcgctgtatggcgagatctttaaggtgccggagccgtttattccgaaatctggcgcgc
gcgtaatgtcgctgctggagccgaccaagaagatgtccaagtctgacgataatcgcaataacgttatcggcctgctggaagatccgaaatcggtagtgaagaaaatcaaacgtgcggtc
actgactccgacgagccgccggtagttcgctacgatgtgcagaacaaagcgggcgtttccaacctgttggatatcctttcagcggtaacgggccagagcatcccagaactggaaaaaca
gttcgaaggcaagatgtatggtcatctgaaaggtgaagtggctgatgccgtttccggtatgctgactgaattgcaggaacgctatcaccgtttccgcaacgatgaagccttcctgcaacaggt
gatgaaagatggcgcggaaaaagccagcgcgcacgcttcccgtacgctaaaagcggtgtacgaagcgattggttttgtggcgaagccgtaagaattcaacgcgttaagtcgactttaact
cgagtctagagggcccgtttaaacccgctgatcagcctcgactgtgccttctagttgccagccatctgttgtttgcccctcccccgtgccttccttgaccctggaaggtgccactcccactgtcctt
tcctaataaaatgaggaaattgcatcgcattgtctgagtaggtgtcattctattctggggggtggggtggggcaggacagcaagggggaggattgggaagacaatagcaggcatgctggg
gatgcggtgggctctatggtaccttaattaagcttggtaccacgcgccctgtagcggcgcattaagcgcggcgggtgtggtggttacgcgcagcgtgaccgctacacttgccagcgccctag
cgcccgctcctttcgctttcttcccttcctttctcgccacgttcgccggctttccccgtcaagctctaaatcgggggctccctttagggttccgatttagtgctttacggcacctcgaccccaaaaaac
ttgattagggtgatggttcacgtagtgggccatcgccctgatagacggtttttcgccctttgacgttggagtccacgttctttaatagtggactcttgttccaaactggaacaacactcaaccctatc
tcggtctattcttttgatttataagggattttgccgatttcggcctattggttaaaaaatgagctgatttaacaaaaatttaacgcgaattttaacaaaatattaacgcttacaatttaggtggcacttttc
ggggaaatgtgcgcggaacccctatttgtttatttttctaaatacattcaaatatgtatccgctcatgagacaataaccctgataaatgcttcaataatattgaaaaaggaagagtatgagtattc
aacatttccgtgtcgcccttattcccttttttgcggcattttgccttcctgtttttgctcacccagaaacgctggtgaaagtaaaagatgctgaagatcagttgggtgcacgagtgggttacatcgaa
ctggatctcaacagcggtaagatccttgagagttttcgccccgaagaacgttttccaatgatgagcacttttaaagttctgctatgtggcgcggtattatcccgtattgacgccgggcaagagca
actcggtcgccgcatacactattctcagaatgacttggttgagtactcaccagtcacagaaaagcatcttacggatggcatgacagtaagagaattatgcagtgctgccataaccatgagtg
ataacactgcggccaacttacttctgacaacgatcggaggaccgaaggagctaaccgcttttttgcacaacatgggggatcatgtaactcgccttgatcgttgggaaccggagctgaatga
agccataccaaacgacgagcgtgacaccacgatgcctgtagcaatggcaacaacgttgcgcaaactattaactggcgaactacttactctagcttcccggcaacaattaatagactggat
ggaggcggataaagttgcaggaccacttctgcgctcggcccttccggctggctggtttattgctgataaatctggagccggtgagcgtgggtctcgcggtatcattgcagcactggggccag
atggtaagccctcccgtatcgtagttatctacacgacggggagtcaggcaactatggatgaacgaaatagacagatcgctgagataggtgcctcactgattaagcattggtaactgtcaga
ccaagtttactcatatatactttagattgatttaaaacttcatttttaatttaaaaggatctaggtgaagatcctttttgataatctcatgaccaaaatcccttaacgtgagttttcgttccactgagcgtc
agaccccgtagaaaagatcaaaggatcttcttgagatcctttttttctgcgcgtaatctgctgcttgcaaacaaaaaaaccaccgctaccagcggtggtttgtttgccggatcaagagctacca
actctttttccgaaggtaactggcttcagcagagcgcagataccaaatactgttcttctagtgtagccgtagttaggccaccacttcaagaactctgtagcaccgcctacatacctcgctctgct
aatcctgttaccagtggctgctgccagtggcgataagtcgtgtcttaccgggttggactcaagacgatagttaccggataaggcgcagcggtcgggctgaacggggggttcgtgcacacag
cccagcttggagcgaacgacctacaccgaactgagatacctacagcgtgagctatgagaaagcgccacgcttcccgaagggagaaaggcggacaggtatccggtaagcggcaggg
tcggaacaggagagcgcacgagggagcttccagggggaaacgcctggtatctttatagtcctgtcgggtttcgccacctctgacttgagcgtcgatttttgtgatgctcgtcaggggggcgga
gcctatggaaaaacgccagcaacgcggcctttttacggttcctggccttttgctggccttttgctcacatgtgcggccg

pIDTsmart-RC2-ΔVP1-Rep,AAP(TAAstop)-456-TAG-CMV-VP1-454-TGA

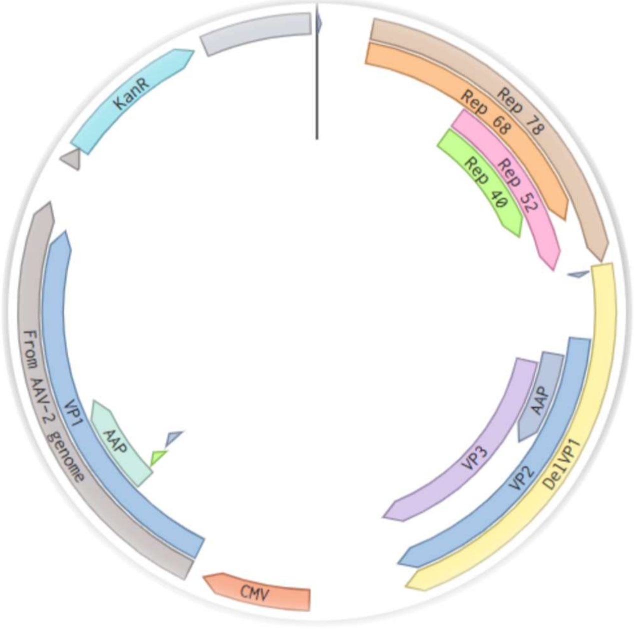

cccgtgtaaaacgacggccagtttatctagtcagcttgattctagctgatcgtggaccggaaggtgagccagtgagttgattgcagtccagttacgctggagtctgaggctcgtcctgaatgat
atgcgaccgccggagggttgcgtttgagacgggcgacagatccagtcgcgctgctctcgtcgatccgctagggcggccgctctagaactagtggatcccccggaagatcagaagttccta
ttccgaagttcctattctctagaaagtataggaacttctgatctgcgcagccgccatgccggggttttacgagattgtgattaaggtccccagcgaccttgacgagcatctgcccggcatttctga
cagctttgtgaactgggtggccgagaaggaatgggagttgccgccagattctgacatggatctgaatctgattgagcaggcacccctgaccgtggccgagaagctgcagcgcgactttctg
acggaatggcgccgtgtgagtaaggccccggaggcccttttctttgtgcaatttgagaagggagagagctacttccacatgcacgtgctcgtggaaaccaccggggtgaaatccatggtttt
gggacgtttcctgagtcagattcgcgaaaaactgattcagagaatttaccgcgggatcgagccgactttgccaaactggttcgcggtcacaaagaccagaaatggcgccggaggcggga
acaaggtggtggatgagtgctacatccccaattacttgctccccaaaacccagcctgagctccagtgggcgtggactaatatggaacagtatttaagcgcctgtttgaatctcacggagcgt
aaacggttggtggcgcagcatctgacgcacgtgtcgcagacgcaggagcagaacaaagagaatcagaatcccaattctgatgcgccggtgatcagatcaaaaacttcagccaggtac
atggagctggtcgggtggctcgtggacaaggggattacctcggagaagcagtggatccaggaggaccaggcctcatacatctccttcaatgcggcctccaactcgcggtcccaaatcaa
ggctgccttggacaatgcgggaaagattatgagcctgactaaaaccgcccccgactacctggtgggccagcagcccgtggaggacatttccagcaatcggatttataaaattttggaacta
aacgggtacgatccccaatatgcggcttccgtctttctgggatgggccacgaaaaagttcggcaagaggaacaccatctggctgtttgggcctgcaactaccgggaagaccaacatcgcg
gaggccatagcccacactgtgcccttctacgggtgcgtaaactggaccaatgagaactttcccttcaacgactgtgtcgacaagatggtgatctggtgggaggaggggaagatgaccgcc
aaggtcgtggagtcggccaaagccattctcggaggaagcaaggtgcgcgtggaccagaaatgcaagtcctcggcccagatagacccgactcccgtgatcgtcacctccaacaccaac
atgtgcgccgtgattgacgggaactcaacgaccttcgaacaccagcagccgttgcaagaccggatgttcaaatttgaactcacccgccgtctggatcatgactttgggaaggtcaccaagc
aggaagtcaaagactttttccggtgggcaaaggatcacgtggttgaggtggagcatgaattctacgtcaaaaagggtggagccaagaaaagacccgcccccagtgacgcagatataag
tgagcccaaacgggtgcgcgagtcagttgcgcagccatcgacgtcagacgcggaagcttcgatcaactacgcagacaggtaccaaaacaaatgttctcgtcacgtgggcatgaatctg
atgctgtttccctgcagacaatgcgagagaatgaatcagaattcaaatatctgcttcactcacggacagaaagactgtttagagtgctttcccgtgtcagaatctcaacccgtttctgtcgtcaa
aaaggcgtatcagaaactgtgctacattcatcatatcatgggaaaggtgccagacgcttgcactgcctgcgatctggtcaatgtggatttggatgactgcatctttgaacaataaatgatttaaa
tcaggtctcgctgccgatggttatcttccagattggctcgaggacactctctctaaaggaataagacagtggtggaagctcaaacctggcccaccaccaccaaagcccgcagagcggcat
aaggacgacagcaggggtcttgtgcttcctgggtacaagtacctcggacccttcaacggactcgacaagggagagccggtcaacgaggcagacgccgcggccctcgagcacgacaa
agcctacgaccggcagctcgacagcggagacaacccgtacctcaagtacaaccacgccgacgcggagtttcaggagcgccttaaagaagatacgtcttttgggggcaacctcggacg
agcagtcttccaggcgaaaaagagggttcttgaacctctgggcctggttgaggaacctgttaagacggctccgggaaaaaagaggccggtagagcactctcctgtggagccagactcct
cctcgggaaccggaaaggcgggccagcagcctgcaagaaaaagattgaattttggtcagactggagacgcagactcagtacctgacccccagcctctcggacagccaccagcagcc
ccctctggtctgggaactaatacgatggctacaggcagtggcgcaccaatggcagacaataacgagggcgccgacggagtgggtaattcctcgggaaattggcattgcgattccacatg
gatgggcgacagagtcatcaccaccagcacccgaacctgggccctgcccacctacaacaaccacctctacaaacaaatttccagccaatcaggagcctcgaacgacaatcactacttt
ggctacagcaccccttgggggtattttgacttcaacagattccactgccacttttcaccacgtgactggcaaagactcatcaacaacaactggggattccgacccaagagactcaacttcaa
gctctttaacattcaagtcaaagaggtcacgcagaatgacggtacgacgacgattgccaataaccttaccagcacggttcaggtgtttactgactcggagtaccagctcccgtacgtcctcg
gctcggcgcatcaaggatgcctcccgccgttcccagcagacgtcttcatggtgccacagtatggatacctcaccctaaacaacgggagtcaggcagtaggacgctcttcattttactgcctg
gagtactttccttctcagatgctgcgtaccggaaacaactttaccttcagctacacttttgaggacgttcctttccacagcagctacgctcacagccagagtctggaccgtctcatgaatcctctc
atcgaccagtacctgtattacttgagcagaacaaacactccaagtggaaccacctagcagtcaaggcttcagttttctcaggccggagcgagtgacattcgggaccagtctaggaactgg
cttcctggaccctgttaccgccagcagcgagtatcaaagacatctgcggataacaacaacagtgaatactcgtggactggagctaccaagtaccacctcaatggcagagactctctggtg
aatccgggcccggccatggcaagccacaaggacgatgaagaaaagtttttcctcagagcggggttctcatctttgggaagcaaggctcagagaaaacaaatgtggacattgaaaaggt
catgattacagacgaagaggaaatcaggacaaccaatcccgtggctacggagcagtatggttctgtatctaccaacctccagagaggcaacagacaagcagctaccgcagatgtcaa
cacacaaggcgttcttccaggcatggtctggcaggacagagatgtgtaccttcaggggcccatctgggcaaagattccacacacggacggacattttcacccctctcccctcatgggtgga
ttcggacttaaacaccctcctccacagattctcatcaagaacaccccggtacctgcgaatccttcgaccaccttcagtgcggcaaagtttgcttccttcatcacacagtactccacgggacag
gtcagcgtggagatcgagtgggagctgcagaaggaaaacagcaaacgctggaatcccgaaattcagtacacttccaactacaacaagtctgttaatgtggactttactgtggacactaat
ggcgtgtattcagagcctcgccccattggcaccagatacctgactcgtaatctgtaattgcttgttaatcaataaaccgtttaattcgtttcagttgaactttggtctctgcgtatttctttcttatctagttt
ccatggctacgtagataagtagcatggcgggttaatcattaactacagcccgggcgtttaaacagcgggcggaggggtggagtcgtgacgtgaattacgtcatagggttagggaggtcct

gtattagaggtcacgtgagtgttttgcgacattttgcgacaccatgtggtctcgctgggggggggggcccgagtgagcacgcagggtctccattttgaagcgggaggtttgaacgagcgctg
gcgcgctcactggccgtcgttttacaacgtcgtgactgggaaaaccctggcgttacccaacttaatcgccttgcagcacatccccctttcgccagctggcgtaatagcgaagaggcccgca
ccgatcgcccttcccatgcatcggccgcaaatacctgcaggatccgttttgcgctgcttcgcgatgtacgggccagatatacgcgttgacattgattattgactagttattaatagtaatcaattac
ggggtcattagttcatagcccatatatggagttccgcgttacataacttacggtaaatggcccgcctggctgaccgcccaacgacccccgcccattgacgtcaataatgacgtatgttcccat
agtaacgccaatagggactttccattgacgtcaatgggtggactatttacggtaaactgcccacttggcagtacatcaagtgtatcatatgccaagtacgccccctattgacgtcaatgacggt
aaatggcccgcctggcattatgcccagtacatgaccttatgggactttcctacttggcagtacatctacgtattagtcatcgctattaccatggtgatgcggttttggcagtacatcaatgggcgtg
gatagcggtttgactcacggggatttccaagtctccaccccattgacgtcaatgggagtttgttttggcaccaaaatcaacgggactttccaaaatgtcgtaacaactccgccccattgacgca
aatgggcggtaggcgtgtacggtgggaggtctatataagcagagctctctggctaactagagaacccactgcttactggcttatcgaaattaatacgactcactatagggagacccaagct
ggctagcatggctgccgatggttatcttccagattggctcgaggacactctctctgaaggaataagacagtggtggaagctcaaacctggcccaccaccaccaaagcccgcagagcggc
ataaggacgacagcaggggtcttgtgcttcctgggtacaagtacctcggacccttcaacggactcgacaagggagagccggtcaacgaggcagacgccgcggccctcgagcacgac
aaagcctacgaccggcagctcgacagcggagacaacccgtacctcaagtacaaccacgccgacgcggagtttcaggagcgccttaaagaagatacgtcttttgggggcaacctcgga
cgagcagtcttccaggcgaaaaagagggttcttgaacctctgggcctggttgaggaacctgttaagaccgctccgggaaaaaagaggccggtagagcactctcctgtggagccagactc
ctcctcgggaaccggaaaggcgggccagcagcctgcaagaaaaagattgaattttggtcagactggagacgcagactcagtacctgacccccagcctctcggacagccaccagcag
ccccctctggtctcggaactaataccctcgctacaggcagtggcgcaccactcgcagacaataacgagggcgccgacggagtgggtaattcctcgggaaattggcattgcgattccacat
ggctcggcgacagagtcatcaccaccagcacccgaacctgggccctgcccacctacaacaaccacctctacaaacaaatttccagccaatcaggagcctcgaacgacaatcactactt
tggctacagcacccccttgggggtattttgacttcaacagattccactgccacttttcaccacgtgactggcaaagactcatcaacaacaactggggattccgacccaagagactcaacttca
agctctttaacattcaagtcaaagaggtcacgcagaatgacggtacgacgacgattgccaataaccttaccagcacggttcaggtgtttactgactcggagtaccagctcccgtacgtcctc
ggctcggcgcatcaaggatgcctcccgccgttcccagcagacgtcttcatggtgccacagtatggatacctcaccctaaacaacgggagtcaggcagtaggacgctcttcattttactgcct
ggagtactttccttctcagatgctgcgtaccggaaacaactttaccttcagctacacttttgaggacgttcctttccacagcagctacgctcacagccagagtctggaccgtctcatgaatcctct
catcgaccagtacctgtattacttgagcagaacaaacactccaagtggatgaaccacgcagtcaaggcttcagttttctcaggccggagcgagtgacattcgggaccagtctaggaactg
gcttcctggaccctgttaccgccagcagcgagtatcaaagacatctgcggataacaacaacagtgaatactcgtggactggagctaccaagtaccacctcaatggcagagactctctggt
gaatccgggcccggccatggcaagccacaaggacgatgaagaaaagtttttttcctcagagcggggttctcatctttgggaagcaaggctcagagaaaacaaatgtggacattgaaaag
gtcatgattacagacgaagaggaaatcaggacaaccaatcccgtggctacggagcagtatggttctgtatctaccaacctccagagaggcaacagacaagcagctaccgcagatgtca
acacacaaggcgttcttccaggcatggtctggcaggacagagatgtgtaccttcaggggcccatctgggcaaagattccacacacggacggacattttcacccctctcccctcatgggtgg
attcggacttaaacaccctcctccacagattctcatcaagaacaccccggtacctgcgaatccttcgaccaccttcagtgcggcaaagtttgcttccttcatcacacagtactccacgggaca
ggtcagcgtggagatcgagtgggagctgcagaaggaaaacagcaaacgctggaatcccgaaattcagtacacttccaactacaacaagtctgttaatgtggactttactgtggacactaa
tggcgtgtattcagagcctcgccccattggcaccagatacctgactcgtaatctgtaattgcttgttaatcaataaaccgtttaattcgtttcagttgaactttggtctctgcgtatttctttcttatctagtt
tccatggctacgtagataagtagcatggcgggttaatcattaactacagccctaggggtgcgagcggatcgagcagtgtcgatcactactggaccgcgagctgtgctgcgacccgtgatctt
acggcattatacgtatgatcggtccacgatcagctagattatctagtcagcttgatgtcatagctgtttcctgaggctcaatactgaccatttaaatcatacctgacctccatagcagaaagtcaa
aagcctccgaccggaggcttttgacttgatcggcacgtaagaggttccaactttcaccataatgaaataagatcactaccgggcgtatttttttgagttatcgagattttcaggagctaaggaag
ctaaaatgagccatattcaacgggaaacgtcttgcttgaagccgcgattaaattccaacatggatgctgatttatatgggtataaatgggctcgcgataatgtcgggcaatcaggtgcgacaa
tctatcgattgtatgggaagcccgatgcgccagagttgtttctgaaacatggcaaaggtagcgttgccaatgatgttacagatgagatggtcaggctaaactggctgacggaatttatgcctctt
ccgaccatcaagcattttatccgtactcctgatgatgcatggttactcaccactgcgatccccagggaaaacagcattccaggtattagaagaatatcctgattcaggtgaaaatattgttgatgc
gctggcagtgttcctgcgccggttgcattcgattcctgtttgtaattgtccttttaacggcgatcgcgtatttcgtctcgctcaggcgcaatcacgaatgaataacggtttggttggtgcgagtgatttt
gatgacgagcgtaatggctggcctgttgaacaagtctggaaagaaatgcataaactcttgccattctcaccggattcagtcgtcactcatggtgatttctcacttgataaccttatttttgacgag
gggaaattaataggttgtattgatgttggacgagtcggaatcgcagaccgataccaggatcttgccatcctatggaactgcctcggtgagttttctccttcattacagaaacggctttttcaaaaa
tatggtattgataatcctgatatgaataaattgcagtttcacttgatgctcgatgagtttttctaatgaggacctaaatgtaatcacctggctcaccttcgggtgggcctttctgcgttgctggcgtttttc
cataggctccgcccccctgacgagcatcacaaaaatcgatgctcaagtcagaggtggcgaaacccgacaggactataaagataccaggcgtttccccctggaagctccctcgtgcgctc
tcctgttccgaccctgccgcttaccggatacctgtccgcctttctcccttcgggaagcgtggcgctttctcatagctcacgctgtaggtatctcagttcggtgtaggtcgttcgctccaagctgggct
gtgtgcacgaaccccccgttcagcccgaccgctgcgccttatccggtaactatcgtcttgagtccaacccggtaagacacgacttatcgccactggcagcagccactggtaacaggattag
cagagcgaggtatgtaggcggtgctacagagttcttgaagtggtggcctaactacggctacactagaagaacagtatttggtatctgcgctctgctgaagccagttacctcggaaaaagagt
tggtagctcttgatccggcaaacaaaccaccgctggtagcggtggtttttttgtttgcaagcagcagattacgcgcagaaaaaaaggatctcaagaagatcctttgatttctaccgaagaaa
ggccca

References:

(1) Kelemen, R. E.; Mukherjee, R.; Cao, X.; Erickson, S. B.; Zheng, Y.; Chatterjee, A. A Precise Chemical Strategy To Alter the Receptor Specificity of the Adeno-Associated Virus. *Angew Chem Int Ed Engl* **2016**, *55* (36), 10645–10649. https://doi.org/10.1002/anie.201604067.

(2) Pham, Q.; Glicksman, J.; Shahraeini, S.; Han, B.; Jewel, D.; Loynd, C.; Roy, S. J. S.; Chatterjee, A. A Facile Chemical Strategy to Synthesize Precise AAV-Protein Conjugates for Targeted Gene Delivery. bioRxiv July 20, 2024, p 2024.07.20.604406. https://doi.org/10.1101/2024.07.20.604406.

(3) Zheng, Y.; Mukherjee, R.; Chin, M. A.; Igo, P.; Gilgenast, M. J.; Chatterjee, A. Expanding the Scope of Single and Dual Noncanonical Amino Acid Mutagenesis in Mammalian Cells Using Orthogonal Polyspecific Leucyl-tRNA Synthetases. *Biochemistry* **2018**, *57* (4), 441–445. https://doi.org/10.1021/acs.biochem.7b00952.

(4) Loynd, C.; Singha Roy, S. J.; Ovalle, V. J.; Canarelli, S. E.; Mondal, A.; Jewel, D.; Ficaretta, E. D.; Weerapana, E.; Chatterjee, A. Electrochemical Labelling of Hydroxyindoles with Chemoselectivity for Site-Specific Protein Bioconjugation. *Nat. Chem.* **2023**, 1–9. https://doi.org/10.1038/s41557-023-01375-y.

(5) Erickson, S. B.; Pham, Q.; Cao, X.; Glicksman, J.; Kelemen, R. E.; Shahraeini, S. S.; Bodkin, S.; Kiyam, Z.; Chatterjee, A. Precise Manipulation of the Site and Stoichiometry of Capsid Modification Enables Optimization of Functional Adeno-Associated Virus Conjugates. *Bioconjugate Chem.* **2024**, *35* (1), 64–71. https://doi.org/10.1021/acs.bioconjchem.3c00411.

(6) Grasso, K. T.; Singha Roy, S. J.; Osgood, A. O.; Yeo, M. J. R.; Soni, C.; Hillenbrand, C. M.; Ficaretta, E. D.; Chatterjee, A. A Facile Platform to Engineer Escherichia Coli Tyrosyl-tRNA Synthetase Adds New Chemistries to the Eukaryotic Genetic Code, Including a Phosphotyrosine Mimic. *ACS Cent. Sci.* **2022**, *8* (4), 483–492. https://doi.org/10.1021/acscentsci.1c01465.

(7) Italia, J. S.; Addy, P. S.; Wrobel, C. J. J.; Crawford, L. A.; Lajoie, M. J.; Zheng, Y.; Chatterjee, A. An Orthogonalized Platform for Genetic Code Expansion in Both Bacteria and Eukaryotes. *Nat Chem Biol* **2017**, *13* (4), 446–450. https://doi.org/10.1038/nchembio.2312.

(8) Jang, H. S.; Jana, S.; Blizzard, R. J.; Meeuwsen, J. C.; Mehl, R. A. Access to Faster Eukaryotic Cell Labeling with Encoded Tetrazine Amino Acids. *J. Am. Chem. Soc.* **2020**, *142* (16), 7245–7249. https://doi.org/10.1021/jacs.9b11520.

(9) Singha Roy, S. J.; Loynd, C.; Jewel, D.; Canarelli, S. E.; Ficaretta, E. D.; Pham, Q. A.; Weerapana, E.; Chatterjee, A. Photoredox-Catalyzed Labeling of Hydroxyindoles with Chemoselectivity (PhotoCLIC) for Site-Specific Protein Bioconjugation. *Angewandte Chemie International Edition* **2023**, *62* (27), e202300961. https://doi.org/10.1002/anie.202300961.
